# Hope in breastfeeding in the context of breastfeeding counseling: a qualitative analysis based on the Multidimensional Model of Hope

**DOI:** 10.1590/1980-220X-REEUSP-2025-0394en

**Published:** 2026-09-04

**Authors:** Allison Scholler de Castro Villas Boas, Monika Wernet, Mariana Torreglosa Ruiz, Aline Oliveira Silveira, Zaida Charepe, Ana Maria Linares, Jéssica Aparecida da Silva

**Affiliations:** 1 Universidade Federal de São Carlos, Programa de Pós-Graduação em Enfermagem, São Carlos, SP, Brazil.; 2 Universidade Federal do Triângulo Mineiro, Programa de Pós-Graduação em Atenção à Saúde, Uberaba, MG, Brazil.; 3 Universidade de Brasília, Brasília, DF, Brazil.; 4 Universidade Católica Portuguesa, Centro de Investigação Interdisciplinar em Saúde, Lisboa, Portugal.; 5 University of Kentucky, College of Nursing, Lexington, United States.

**Keywords:** Breast Feeding, Hope, Counseling, Weaning, Qualitative Research

## Abstract

**Objective:**

To understand hope in breastfeeding from the perspective of women who received breastfeeding counselling.

**Method:**

A qualitative study nested within a clinical trial, based on thematic analysis guided by the Multidimensional Model of Hope. The study included 11 women interviewed between 14 and 18 months postpartum, all of whom had participated in the pilot study of a clinical trial and had received breastfeeding counselling in rooming-in units of maternity hospitals located in Minas Gerais, Rio de Janeiro, and Bahia, Brazil. A single, semi-structured, audio-recorded interview was conducted between May and September 2024.

**Results:**

Women perceived breastfeeding as an act that positively affects child health and promotes mother–child bonding, both regarded as expected outcomes. Breastfeeding counselling was essential in helping them overcome doubts, fears, and breastfeeding-related challenges. This supportive relationship constituted an experience that activated dimensions of hope and left lasting emotional memories.

**Conclusion:**

The relationship with the breastfeeding counsellor, a component of the affiliative dimension of hope, facilitated and structured women’s coping with breastfeeding challenges toward the hoped-for outcome.

## INTRODUCTION

The World Health Organization (WHO)^(1)^ and the Brazilian Ministry of Health^(2)^ recommend exclusive feeding with human milk (HM) as the best way to nourish infants during the first six months of life. Despite these recommendations, exclusive breastfeeding rates remain below the recommended levels^(3)^, with frequent use of supplements and infant formula in Brazil and worldwide^(4)^. Fewer than half of all infants are exclusively breastfed until six months of age, although the WHO recommends efforts to increase the prevalence of exclusive breastfeeding to 60% by 2030^(5)^.

Investments in the promotion and protection of breastfeeding are urgently needed, and breastfeeding counselling (BC) represents one such strategy. BC is grounded in acknowledging and respecting breastfeeding women, their wishes, and their individual possibilities regarding breastfeeding^(6)^. The counsellor places the encounter and dialogue at the center of breastfeeding support, requiring strong interpersonal and communication skills^(6)^.

A systematic review with meta-analysis demonstrated that BC is effective in increasing the duration of exclusive breastfeeding^(7)^. Evidence indicates that BC increases the likelihood of exclusive breastfeeding throughout the first six months of life^(8)^ and influences both the decision to breastfeed and breastfeeding maintenance during the child’s first year of life^(9)^.

The belief that HM is beneficial for the child^(9,10)^, together with social support networks—particularly emotional and practical support for breastfeeding^(11)^—are determining factors in both the decision to breastfeed and breastfeeding maintenance. Information, cultural beliefs, and personal and family values also influence breastfeeding decisions^(9,10)^.

However, although public policies and institutional discourse promote breastfeeding, insecurity, difficulties, pain, and loneliness are common experiences among breastfeeding women^(12)^, while social and cultural pressures often shape infant feeding decisions, interfering with breastfeeding plans. Within this context, hope emerges as a resource that facilitates coping and recovery^(13)^, with evidence suggesting that it can be fostered.

Hope has increasingly been recognized as an important component of healthcare and nursing. Considering hope in the context of breastfeeding is an innovative approach with the potential to stimulate discussion, particularly because it encompasses individual characteristics, desires, and subjectivities^(14)^. Furthermore, it may provide valuable insights for healthcare and nursing practice, including within the context of BC. To date, the breastfeeding literature has not addressed hope as a central topic. No qualitative studies were identified that examined hope within the context of BC. Therefore, the following research question was proposed: How do women who received BC in the rooming-in unit (RIU) experience hope? Accordingly, this study aimed to understand hope in breastfeeding from the perspective of women who received BC.

## METHOD

### Study Design

The COnsolidated criteria for REporting Qualitative research^(15)^ guided the reporting of the study and the writing of this article.

This was a qualitative study based on the Multidimensional Model of Hope^(16)^, which conceptualizes hope through two spheres (generalized and particularized), each comprising the same six dimensions (affective, cognitive, affiliative, behavioral, contextual, and temporal)^(16)^. The particularized sphere differs from the generalized sphere in that the hoped-for outcome is considered attainable, thereby promoting constructive coping^(16)^.

According to the model adopted in this study, the contextual dimension acknowledges the influence of life circumstances on hope. It is related to the temporal dimension, which recognizes the influence of individuals’ life histories and the interconnectedness of the past, present, and future. These dimensions interact with the cognitive dimension, which encompasses desires, intentions, knowledge, and the appraisal of life events and determining factors. Sensations, emotions, and feelings comprise the affective dimension, whereas actions and behaviors directed toward managing and achieving the hoped-for outcome constitute the behavioral dimension. Social interactions (with oneself and with others) and their implications for lived experience are part of the affiliative dimension^(16)^.

### Setting, Population, and Selection Criteria

This study was nested within the pilot study of a randomized clinical trial (RCT), conducted between February and June 2023 in three RIUs of university hospital maternity units located in Minas Gerais, Rio de Janeiro, and Bahia, Brazil.

The participants belonged to the intervention group of the RCT pilot study, which aimed to investigate the effectiveness of BC during the mother–infant dyad’s stay in the RIU^(17)^. The intervention group received two to four BC sessions during hospitalization in the RIU, delivered by a nurse counsellor trained in BC (76 hours of theoretical and practical training). It is noteworthy that the researchers involved in the present study did not participate in either the intervention or the data collection of the RCT pilot study.

The study included primiparous women who intended to breastfeed, had participated in the intervention group of the RCT pilot study described above, and had the personal and technical resources (particularly internet access) required to participate in an online narrative interview.

All 29 women who participated in the intervention group of the pilot study were invited to participate. Invitations were extended during follow-up telephone calls conducted as part of the pilot study and reinforced through written messages sent via WhatsApp®. Women who agreed to participate received an online informed consent form and scheduled the interview at a date and time of their preference. Of the 29 women invited, six declined to participate (three because they lacked the necessary resources to participate in video calls and three because they did not have time for the interview), while 12 did not respond after three contact attempts by the researchers. Thus, 11 women participated in the present study.

### Data Collection

Data were collected between May and September 2024 through a single, individual, semi-structured online interview conducted via WhatsApp® video calls and audio-recorded using a digital recorder. At the beginning of each video call, participants were asked for permission to record the interview, and the purpose of the recording was clearly explained. The interviewers, who were the first and last authors of this manuscript, kept their cameras on throughout the interviews to facilitate interaction. Participants were free to choose whether to keep their own cameras on or off, according to their comfort.

The interviewers received 20 hours of training in qualitative interviewing, provided by the second author, a researcher with expertise in qualitative research. Interviews lasted between 26 and 52 minutes (mean: 30 minutes) and were initiated with the prompt: “Tell me how you have been feeding your child from birth to the present day”. This was followed by the questions: “Think back to your interactions with the breast­feeding counsellor. What was meaningful to you?” and “How did these interactions influence your hope and your decision-making regarding breastfeeding?” Additional questions were asked to deepen understanding of participants’ narratives.

By the ninth interview, the researchers observed that the dataset already allowed a comprehensive understanding of the experience of hope, with recurring structural elements of the phenomenon indicating sufficient data richness^(18)^. Since only two women who had expressed interest in participating had not yet been interviewed, these interviews were also conducted, resulting in a final sample of 11 participants.

### Data Analysis and Processing

The analytical process began with the preparation of research notes by the first and last authors of this manuscript following each interview. These notes supported the interpretations and inferences developed during the analysis and discussion of empirical data. To minimize the influence of individual bias and ensure the trustworthiness of the analysis, investigator triangulation was adopted. The first and second authors independently conducted the stages of data analysis, with support from researchers with expertise in both the study theme and the Multidimensional Model of Hope (the fourth, fifth, and sixth authors of this manuscript).

The audio recordings were stored in a Google Drive folder belonging to the first and last authors until they were fully transcribed. The interviews were transcribed using the Transkriptor® application and transferred to a Microsoft Word® document by the first author, after which they were thoroughly reviewed by the second author. Once verified by the researchers, the transcripts were sent to the participants via WhatsApp® so that they could suggest corrections, if necessary. No revisions were requested; therefore, the transcripts were considered validated. Following validation, the audio recordings were permanently deleted from cloud storage.

Thematic analysis^(19)^ was adopted as the methodological framework and was conducted by the first author under the supervision of the last author. Initially, the interview transcripts and research notes were read repeatedly to identify meanings and patterns associated with breastfeeding, hope, and BC. Representative excerpts (selection of key elements) were extracted and organized using Atlas.ti® software to facilitate thematic grouping. These thematic groupings subsequently underwent further analysis guided by the Multidimensional Model of Hope^(16)^. This process sought to understand the object of hope, its manifestations, feelings, relationships, and the articulation of these elements within the process of hope in breastfeeding and BC. The analysis also sought to understand how temporality and historicity—encompassing the past, present, and future—were expressed and influenced hope in breastfeeding. Codes were identified and subsequently grouped into themes (code selection and theme development stages). The patterns and relationships among these themes (conceptualization stage) were examined and led to the development of the conceptual model, the final stage of the method^(19)^. Thus, a theory-driven analysis was employed.

Regarding reflexivity and positionality, the researchers responsible for data collection and analysis shared several characteristics: they were maternal and child health nurses, believed in the benefits of HM, and supported breastfeeding. They differed, however, in whether they were mothers themselves. Positionality was addressed during the qualitative interview training described above, which contributed to minimizing its potential influence throughout data collection and analysis.

Throughout the research process, the researchers continuously reflected on and critically examined their own reflexivity and positionality through analytical discussions, reflective field notes recorded during data collection and analysis, and critical review of the emerging categories. These strategies were intended to prevent assumptions favorable to breastfeeding from shaping the interpretation of participants’ narratives uncritically, thereby ensuring openness to expressions of ambivalence, difficulties, or negative experiences reported by the participants. This process allowed the meanings women attributed to their experiences to be understood in all their complexity, preserving the centrality of participants’ voices while reducing the risk of reinforcing normative pro-breastfeeding narratives.

### Ethical Aspects

This study received ethical approval under Opinion 6,274,311, issued on August 31, 2023 (Certificate of Presentation for Ethical Consideration 61321122.3.1001.8667), and complied with the Brazilian National Health Council Resolution 510/2016, which establishes the ethical guidelines and regulatory standards for research involving human participants. All participants signed an informed consent form. In the excerpts presented, participants are identified by the letter “W”, referring to “woman”, followed by the ordinal number corresponding to the order in which they entered the study.

## RESULTS

The study included 11 women: four from Minas Gerais, four from Rio de Janeiro, and three from Bahia. All children had been breastfed at some point in their lives, and most (n = 7) were exclusively breastfed for six months; of these, six were still being breastfed at the time of the interview. Eight participants self-identified as mixed-race, ten had more than nine years of education, and six had delivered by cesarean section. At the time of data collection, children ranged in age from 14 to 18 months, with a mean age of 15 months.

The experience of hope in breastfeeding is presented through two themes: “Child health and development: the hoped-for goal of breastfeeding” and “Practical management of breastfeeding and the maintenance of hope”. Chart 1 presents these themes and their corresponding subthemes.

**Chart 1:** Coding of themes and subthemes derived from study data analysis – São Carlos, SP, Brazil, 2025.

Themes	Subthemes
Child health and development: the hoped-for goal of breastfeeding	Recognition of the benefits of human milk
	Taking ownership of breastfeeding
Practical management of breastfeeding and the maintenance of hope	Empathy and judgment from members of the social network
	Facing difficulties through altruism
	The uniqueness of the mother–child relationship during breastfeeding
	Mutuality in the relationship with the counselor

A desire was identified among women to breastfeed their children, in the hope of ensuring the best nutrition, fostering strong bonds, and promoting health and development. Moral altruism proved central to maintaining hope regarding breastfeeding and to processing the sacrifices and “emotional costs” associated with breastfeeding decisions. They were constantly challenged to reshape that hope.

### Theme: Child Health and Development: The Hoped-for Goal of Breastfeeding

All participants hoped to breastfeed because they “recognized the benefits of HM” for their children, which led them to “take ownership of breastfeeding”, a behavior driven and sustained by their moral commitment to their child and by their social role as a breastfeeding mother. They also emphasized the contribution of other breastfeeding women in helping them cope with distress and breastfeeding-related difficulties. The sharing of knowledge and experiences by these women shaped their internal dialogues, increasing their autonomy and providing insights that supported successful breastfeeding.

### Subtheme: Recognition of The Benefits of Human Milk

HM was recognized as the best food for promoting children’s health, motivating women to make every effort to ensure that their children received it: *I said I didn’t want to give formula because I know how essential breastfeeding is for overall development* (W7 – weaned at six months); [...] *there is nothing like breast milk; the child grows up healthy* (W3 – breastfeeding (14 months), EBF until six months); [...] *what kept me from giving up on breastfeeding* [...] *was the importance of the milk—the health benefits it offers and how it prevents disease* (W4 – currently breastfeeding (14 months); exclusively breastfed until six months).

Intersectionalities underpinned the search: [...] *the milk comes for free. The cost is minimal* [...] *I am a single mother, so I chose exclusive breastfeeding partly for economic reasons, in addition to the benefits it offers the child* (W5 – currently breastfeeding (16 months); exclusively breastfed until six months).

This subtheme reflects the cognitive and behavioral dimensions of hope, structuring both the pursuit of and persistence in breastfeeding. Aspects of broader socialization also relate to the contextual, temporal, and affiliative dimensions of hope, all of which were mobilized to sustain hope throughout the breastfeeding experience.

### Subtheme: Taking Ownership of Breastfeeding

The need to take ownership of breastfeeding and expand both theoretical and practical knowledge intensified after the child’s birth: *I wanted to learn more, despite everything I had already seen during pregnancy. Once the baby girl was born, I wanted to learn even more so I would be better prepared* (W3 – breastfeeding (14 months), EBF until the sixth month). The child’s tangible presence and the interactions established with the infant reinforced the intention to provide HM through breastfeeding, reflecting a clear sense of moral altruism.

The complexity and multifactorial nature of breastfeeding were acknowledged, leading women to continually reassess their hope: [...] *because every mother has a different breastfeeding story* [...] *some had a very smooth breastfeeding experience, others had a short period,* [...] *there are babies who end up not latching on,* [...] *and I kept wondering if it would be like that for me too* (W8 – weaned at two months).

Participants faced adversity with support from healthcare professionals and people close to them, especially family members. These relational experiences were recalled through internalized conversations that fostered inner strength and hope: *It was really important that I tried. Because in the midst of that challenge—being a first-time mom, the postpartum period—*[...] *I made a commitment to myself: I’m not going to switch to formula* [...] *having that support from the nurses and so on, I told myself, “We’ll get through this”. It was a very difficult 15 days. My breasts were sore, but I said, “Let’s keep going”* (W7 – weaned at six months).

Breastfeeding, as a future-oriented project, was built in the present through the continual reshaping of hope, involving the cognitive, contextual, temporal, affiliative, and affective dimensions of hope.

### Theme: Practical Management of Breastfeeding and The Maintenance of Hope

This theme encompasses the effects of “empathy and judgment from members of the social network” on breastfeeding decisions. It also addresses the sacrifices women made and the need to “face difficulties through altruism” in managing breastfeeding, an experience sustained by both “the uniqueness of the mother–child relationship during breastfeeding” and “mutuality in the relationship with the counselor”, both of which played important roles in helping women cope with challenges and maintain hope within the context of their individual circumstances.

### Subtheme: Empathy and Judgment from Members of The Social Network

Social relationships, as well as women’s relationships with themselves, were central determinants of hope. Participants simultaneously described experiences of empathy and pride, alongside judgment, distress, and self-sacrifice. These experiences involve the contextual and affiliative dimensions of hope and contributed to the development of particularized hope, in contrast to generalized hope.

Establishing relationships that encouraged and sustained their commitment to breastfeeding helped participants cope with obstacles, particularly during the early postpartum period. The explicit and implicit support of their partners and other significant people was especially valued: *He* (referring to her partner) *saw how much I was struggling with breastfeeding and stood by me* [...] *he kept saying it would pass, and I held onto that thought* (W3 – breastfeeding (14 months), EBF until six months); *Mom, I don’t think I can handle this* [...] *I’m going to the pediatrician and asking him to prescribe formula* [...] *then she said, “You’re going to give formula when you have enough milk? Just bear the pain; think about how you’re going through this out of love for her”* (W4 – breastfeeding (14 months), EBF until six months); [...] *she* (the participant’s mother) *said she felt my pain when my nipple became* raw (W9 – breastfeeding (17 months), EBF until the sixth month). The nursing mother’s mother stood out for her words of encouragement and expressions of sisterhood.

Concurrently, they navigated interactions that discouraged them from moving toward breastfeeding: *Some friends would say things like, “You’re making yourself suffer—just give her formula; every baby drinks formula”* (W7 – weaned at six months); *Oh, that little girl is hungry* [...] *just go buy some milk* [...] *I started getting anxious; I ended up not going to the pediatrician and started supplementing with formula on my own* [...] *I gave her a bottle, and she started rejecting the breast* (W11 – weaned at two months).

### Subtheme: Facing Difficulties Through Altruism

Difficulties in the practical management of breastfeeding led participants to reconsider the belief that breastfeeding is an instinctive and natural process free of obstacles: *In my mind, you know, breastfeeding was going to be this beautiful thing. That’s how I pictured it. When it didn’t turn out that way, I felt really bad, because I wanted it so much* […] (W2 – weaning in the first month).

The inadequate presentation—or lack of focus—regarding the difficulties, problems, and challenges associated with breastfeeding was called out, serving as a catalyst for hope and for coping with the situation: *I see people romanticizing breastfeeding as if it were just pure bliss—and it isn’t! It really isn’t! It has its challenges; there are difficult moments... you don’t see the woman with dark circles under her eyes, or the woman staying up in the middle of the night... in the videos they show, the baby is always asleep, the house is always tidy, the child never cries... so, it’s complicated, because we see so much of that, but in reality, it’s not quite like that* (W4 – currently breastfeeding (14 months); exclusively breastfed until six months).

At the beginning of breastfeeding, painful nipple and areolar injuries caused considerable distress and made participants want to give up, requiring them to endure substantial physical and emotional distress: *The first fifteen days were really tough. When it was time for her to nurse, I could already feel it coming... because my breasts hurt... I won’t lie, I felt like giving up* (W3 – currently breastfeeding (14 months), exclusively breastfed until six months); *When* [child’s name] *was about 40 days old, my nipples cracked—both of them* [...] *when it cracks, it hurts, it bleeds* [...] *you feel that sense of desperation* [...] *I suffered for a month* [...] *but I didn’t give up; my daughter nursed through the blood and pain while I cried and bit down on a towel* (W4 – breastfeeding (14 months), EBF until the sixth month). Within the tension between giving up and persisting, moral altruism directed toward providing the best possible nourishment for their children remained central.

Pain and sleep deprivation were frequently reported as negative experiences that participants justified to themselves as sacrifices made for the benefit of their children. This understanding motivated them to persevere: *My breast was sore, but I didn’t care. Sometimes I didn’t even feel the pain* [...] *what I knew was that it was good for my daughter. The importance of breast milk in my daughter’s life and the benefits it could bring later on, when she was older, because she had been breastfed* (W11 – weaned at two months); *To this day, I haven’t managed to get a full night’s sleep* [...] (W7 – weaned at six months).

Self-care, leisure activities, and work were relegated to a secondary role: *I’ve neglected my own self-care because I don’t have time for anything* [...] (paused studies and outings) *because I didn’t want to leave him alone; I’m afraid he’ll go hungry without the breast* (W1 – breastfeeding (14 months), mixed feeding since the fourth month); [...] *I even asked to leave my formal employment during the pregnancy so I could stay as close to him as possible, to keep him from weaning too soon* (W8 – weaned at two months).

Returning to work, postpartum complications, and the child’s hospitalization imposed limitations on breastfeeding and led participants either to revise their breastfeeding plans or to discontinue breastfeeding: *I was very angry* (introduction of a bottle and pacifier by the grandmother), *but in the end, I didn’t say anything because I needed help so I could work* (W1 – breastfeeding (14 months), mixed feeding since the fourth month); [Child’s name] *developed bronchiolitis and had to be hospitalized* [...] *we spent 18 days in isolation; he forgot how to nurse and had to be fed via a tube* [...] *I was the one who kept insisting; I didn’t want to give up* [...] (W8 – weaned at two months).

Regardless of how long they breastfed, all participants sought to resist circumstances that favored weaning: *What I really wanted was to still be breastfeeding now* (at one year and five months). *Unfortunately, he ended up weaning, but I really cherished the time—I have no regrets to this day about everything I went through* (W8 – weaned at two months); *Even though it was mixed feeding, it was quite a struggle for us* [...] *It wasn’t for lack of effort* [...*] I saw it as a worthwhile effort* (W11 – weaned at two months).

The expectation of a breastfeeding experience free from difficulties—often romanticized in social media and online videos—contrasted sharply with participants’ lived experiences. Anchored primarily in the cognitive dimension (knowledge of the benefits of HM) and the affective dimension (the emotional bond with the child), they remained motivated to sustain hope throughout their breastfeeding journey.

### Subtheme: The Uniqueness of The Mother–Child Relationship During Breastfeeding

Women developed and strengthened a unique connection with their children, an experience that gave meaning to breastfeeding and continually reshaped their hope*: It’s a unique moment that belongs only to us* (her and the child) [...] *seeing his little eyes, the happy way he looks at me when I breastfeed him, the way he calms down* (W1 – breastfeeding (14 months), mixed feeding since the fourth month); *It’s something... inexplicable* [...] *him looking at me, placing his little hand, stroking me. So, I loved it* (W9 – breastfeeding (17 months), EBF until the sixth month).

The affective and affiliative dimensions of hope were expressed through love, pleasure, bonding, interaction, and connection with the child, broadening the meaning of breastfeeding beyond the health benefits of HM.

### Subtheme: Mutuality in The Relationship with THE Counselor

BC was perceived as a source of support, particularly during the early stages of breastfeeding. The counselor’s approach helped women cope with fear, distress, and doubts, providing reassurance: *She* (the counselor) *was absolutely essential to my breastfeeding journey. If it weren’t for her, I might not have managed it* (W1 – breastfeeding (14 months), mixed feeding since the fourth month); *Just knowing you aren’t alone in that, that there’s someone there trying to help you* (W2 – weaning in the first month); *I said to* (counselor’s name) [...] *I don’t have any milk. And she said, “No, don’t worry, you do have milk”—and that is so reassuring* (W7 – weaned at six months).

The counselor mediated understanding and provided practical interventions tailored to each breastfeeding woman: [...] *that initial guidance made all the difference* [...] *because, in practice, if you don’t have someone there to advise and assist you, it just doesn’t work out* (W4 – breastfeeding (14 months), EBF until six months); *It was my first experience as a mother, and I was feeling apprehensive at the time; it really helped me clear up my doubts* (W11 – weaned at two months); *I have an inverted nipple, so I was also having some trouble getting the baby to latch. But with her guidance, I managed to get my daughter to breastfeed* (W11 – weaned at two months).

The counselor’s presence and communication were distinguishing features of the experience and remained in participants’ memories: *I will always remember her just like that. I think that the day I have another child, I’ll immediately think of her—specifically regarding breastfeeding* (W3 – currently breastfeeding (14 months); EBF until six months). *My decision to go ahead with breastfeeding stemmed from a conversation—something that really left a mark on me. So, the way she offered support* [...] *changed my entire dynamic with my daughter* (W7 – weaned at six months).

**Figure 1:**
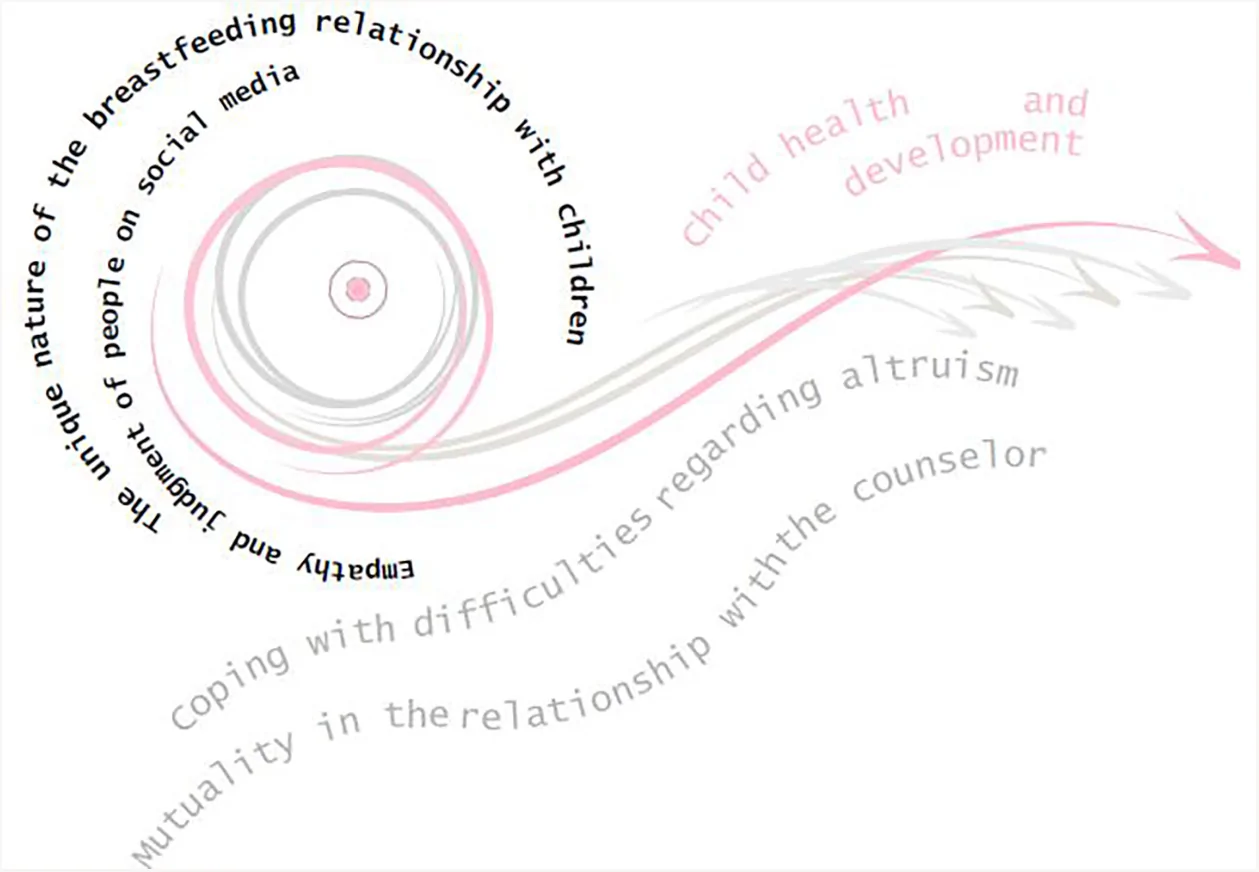
Conceptual model of hope in breastfeeding: the experience of women who received breastfeeding counseling.

Figure 1 presents the conceptual model, together with the themes and subthemes identified in this study. The desire to breastfeed is closely linked to the goal of promoting the child’s health. This process involves “facing difficulties through altruism”, while also encompassing “empathy and judgment from members of the social network”. Throughout this process, “the uniqueness of the mother–child relationship during breastfeeding” becomes evident and is sustained by “mutuality in the relationship with the counselor”. The conceptual model broadens understanding and enables the practical application of the Multidimensional Model of Hope to the specific experience of breastfeeding.

## DISCUSSION

Breastfeeding was conceived as something that positively impacts the child’s health and promotes bonding with the child, both of which were desired and expected. Accordingly, the women committed themselves to breastfeeding in the hope of achieving it while facing its challenges. Hope promoted changes in meanings and pathways throughout the pursuit of maintaining breastfeeding. Specifically, the counselor promoted particularized hope by intervening primarily in the cognitive and affective dimensions of hope, thereby becoming an element of the affiliative dimension of hope.

The literature supports the relationship between breastfeeding, child health, and bonding^(20)^, as well as the urgent need for informational support, whether individual, collective, or broader, such as that provided through breastfeeding campaigns. This study showed that informational support affects hope in breastfeeding by promoting motivation and engagement and acting on agency thinking^(21)^. BC provided highly individualized support and was essential for hope in breastfeeding.

Contexts of uncertainty, in which control is threatened, such as breastfeeding, require hope to promote coping strategies aligned with specific intervening factors. Taking ownership of breastfeeding, a behavior sought by the participants, reflected the pursuit of control and the intention to achieve self-efficacy. The act of taking ownership involves the cognitive, affiliative, behavioral, and contextual dimensions of hope^(16)^.

One way healthcare professionals can facilitate this process is by encouraging pregnant women and breastfeeding women to share their breastfeeding experiences, thereby placing possibilities and contexts into perspective. Listening to these experiences promotes contact with those involved in breastfeeding and makes it possible to address their experiences^(22)^, while recognizing the uniqueness of each breastfeeding experience^(23)^. Professional mediation should address everyone involved in breastfeeding, making their concerns, doubts, and uncertainties tangible while considering the particularities of each situation. Particularization is essential for constructive coping because it allows for critical and contextualized appraisal^(16)^.

Furthermore, curating and recommending websites, videos, and other digital media on breastfeeding is a promising strategy because it facilitates taking ownership of the topic and helps counter the dissemination of romanticized social constructions, which were identified as barriers to breastfeeding.

The practical management of breastfeeding finds an important resource in the empathy of members of the social support network. Family-centered care highlights the urgent need for healthcare professionals to recognize the family as also requiring care, as well as the influence of family dynamics on individual coping processes and vice versa^(24)^, a finding reinforced in this study. Recognizing and considering the family—particularly the individuals who are significant to each breastfeeding woman—is an intervention that promotes hope in breastfeeding. Intra-family relationships strengthen hope when they are grounded in trust, supportive efforts, and attentive listening^(24)^.

The influence of the belief in “weak milk” and questions regarding whether the effort required for breastfeeding is worthwhile were identified and corroborate previous studies^(25)^, revealing the persistence of social constructions and the difficulty in challenging them. Beliefs belong to the cognitive and contextual dimensions and strongly affect the affective and cognitive dimensions of hope^(16)^. They are also strongly associated with weaning^(26)^.

Difficulties and distress were present, and coping occurred amid emotional vulnerability, insecurity, and physical pain, especially pain resulting from nipple-areolar complex injuries, issues that, according to the participants, are rarely or insufficiently addressed in professional breastfeeding support. Advancing approaches focused on the promotion and protection of breastfeeding is essential^(14)^. In this regard, using the Multidimensional Model of Hope in breastfeeding support interventions helps identify and intervene in the factors involved, address meanings, and reshape behaviors.

Within the empathy–altruism gap lie the motivational concepts of altruism and egoism, particularly exclusive egoism and the effects of empathic concern on the development of altruistic motivation^(27)^. Empathic concern refers to an emotion directed toward another person, elicited by and congruent with the perceived well-being of that individual. Altruism, within the egoism–altruism framework, refers to a motivational state whose ultimate goal is to increase another person’s well-being. In this context, altruistic motivation, induced by empathy, is part of human parenthood. Empathy-induced altruism is neither inherently positive nor negative; it simply exists^(27)^, and it was present in this study.

Work, postpartum complications, and the child’s hospitalization affected breastfeeding and gave it particular characteristics. However, none of these situations constitute justification for weaning, although they commonly lead to it^(28)^. For all of these situations, therapeutic care plans involving the Human Milk Bank are essential^(29)^, although they did not emerge in the findings of this study. Future studies examining the relationship between this service and hope in breastfeeding are recommended.

The contributions of breastfeeding to bonding and attachment have been widely described^(20)^, were perceived by the participants, and sustained their hope in breastfeeding. Based on this finding, discussing the relationship with the child during breastfeeding may reveal and validate nuances of this relationship, highlighting eye contact and touch, both of which were reinforced in our findings as promoters of hope. Furthermore, the conceptual model developed in the present study shares similarities with the Interactive Theory of Breastfeeding^(23)^, particularly regarding the goal of achieving breastfeeding and the centrality of interaction in the pursuit of breastfeeding. In other words, women interact with their children and with other interpersonal and social systems in the direction of breastfeeding. Meanings, beliefs, and memories related to their social context and life history are mobilized. This study adds to the aforementioned model^(23)^ evidence of the strength of the construct of hope and its dimensions in understanding the experiences of those involved and their coping process.

Participants emphasized that they received BC and highlighted its contributions to several dimensions of hope, especially the affiliative, cognitive, behavioral, and affective dimensions. However, repercussions were also observed for the contextual and temporal dimensions. The principles of BC, which emphasize dialogue and person-centeredness, are consistent with the foundations of the intentional use of hope in healthcare interventions. The emergence of feelings of being welcomed and of gratitude as outcomes of this relationship provides evidence of this and is consistent with comprehensive, humanized, equitable, and hope-promoting care.

The limitations of this study are related to the small number of participants, all of whom were primiparous women, from three hospital centers, which does not allow the generalization or transferability of the findings. However, qualitative studies are not intended for that purpose. Furthermore, because the participants agreed to take part in the pilot RCT of BC, it may be inferred that they were already inclined to breastfeed. Another limitation is that all participants belonged to the intervention group, that is, they received BC, which may have introduced “gratitude” bias toward the counselor, as well as social desirability bias regarding breastfeeding. Together, these issues represent operational and analytical limitations. On the other hand, it should be emphasized that the objective was to understand hope in breastfeeding, which directed the researchers’ focus toward hope. Nevertheless, studies involving women who discontinued breastfeeding early, did not receive BC, or had no intention or inclination to breastfeed are recommended to further advance the evidence.

Conducting the interviews remotely may also be considered a limitation because it reduced access to nonverbal cues. The importance of conducting future studies using face-to-face interviews should therefore be emphasized. However, it should also be noted that the remote format facilitated participants’ involvement.

Another limitation of the study is the possibility of recall bias, since the interviews were conducted approximately 15 months after the women had received BC. The time elapsed between the intervention and the interview may have compromised the interpretive richness of participants’ memories. Nevertheless, this limitation is believed to have been minimized by the depth and richness of the participants’ narratives.

As a contribution of this study, the narratives showed that hope supports breastfeeding and that counseling sustains hope. Hope organizes the process of coping with breastfeeding; the hoped-for object is projected onto the child rather than the woman; distress is morally legitimized through altruism; and the relationship with the counselor, a component of the affiliative dimension of hope, favored and organized coping with breastfeeding challenges in the direction of the hoped-for object.

## CONCLUSION

Breastfeeding is related to the desire to provide the child with a healthy life and to establish unique and lasting bonds and connections with the child. This desire motivates the pursuit of breastfeeding, hope in breastfeeding, coping with its challenges, and persistence throughout the process.

To achieve greater success in breastfeeding, the participants sought to take ownership of breastfeeding; however, they denounced the romanticized discourse surrounding breastfeeding, which reverberates negatively, particularly in the affective dimension of hope. Furthermore, within the affiliative dimension, they emphasized the importance of integrating the judgments of the social network and empathy. Among other aspects, they managed beliefs, painful experiences, and personal sacrifices in order to breastfeed. In their closest relationships, especially with their partner/the child’s father and with their mothers, they found acceptance and support, as they also did with the counselor. These relationships were combined with the unique connection established with the child, which plays a distinctive role in hope for breastfeeding and is central to mobilizing coping throughout the numerous situations experienced during the breastfeeding journey.

Based on the findings, the following recommendations are proposed to promote hope in breastfeeding: exploring the relationship with the child and the family support network; curating educational and breastfeeding care technologies while avoiding the romanticization of the breastfeeding process; promoting dialogue about work and breastfeeding; investing in collective educational and care strategies; and encouraging the participation of significant partners during prenatal care. Above all, however, investment is needed in the highly individualized relationship with the breastfeeding woman and her family, based on the conceptual model of hope in breastfeeding developed in this study.

## Data Availability

The entire dataset supporting the results of this study is available upon request to the corresponding author.
