# Supplementary material for: Hope in breastfeeding in the context of breastfeeding counseling: a qualitative analysis based on the Multidimensional Model of Hope
Source: Rev Esc Enferm USP. 2026 Sep 4;60:e20250394. doi: 10.1590/1980-220X-REEUSP-2025-0394en (PMC13544913; doi:10.1590/1980-220X-REEUSP-2025-0394en)
Supplement: Translation

# Translation: Esperança na amamentação diante do aconselhamento em aleitamento: análise qualitativa à luz do Modelo Multidimensional da Esperança

**DOI:** 10.1590/1980-220X-REEUSP-2025-0394pt

**Authors:** Allison Scholler de Castro Villas Boas, Monika Wernet, Mariana Torreglosa Ruiz, Aline Oliveira Silveira, Zaida Charepe, Ana Maria Linares, Jéssica Aparecida da Silva

**Affiliations:** 1 Universidade Federal de São Carlos, Programa de Pós-Graduação em Enfermagem, São Carlos, SP, Brasil.; 2 Universidade Federal do Triângulo Mineiro, Programa de Pós-Graduação em Atenção à Saúde, Uberaba, MG, Brasil.; 3 Universidade de Brasília, Brasília, DF, Brasil.; 4 Universidade Católica Portuguesa, Centro de Investigação Interdisciplinar em Saúde, Lisboa, Portugal.; 5 University of Kentucky, College of Nursing, Lexington, Estados Unidos.

**Keywords:** Aleitamento Materno, Esperança, Aconselhamento, Desmame, Pesquisa Qualitativa

## Abstract

**Objetivo:**

Compreender a esperança na amamentação na perspectiva de mulheres que receberam aconselhamento em aleitamento materno.

**Método:**

Pesquisa qualitativa aninhada a um ensaio clínico, embasada na análise temática guiada pelo Modelo Multidimensional da Esperança. Participaram do estudo 11 mulheres, com idade entre 14 e 18 meses após o parto, todas integrantes do estudo piloto de um ensaio clínico, que receberam aconselhamento sobre aleitamento materno no alojamento conjunto de maternidades localizadas em Minas Gerais, Rio de Janeiro e Bahia, no Brasil. Foi conduzida uma entrevista semiestruturada, única e áudio gravada entre maio e setembro de 2024.

**Resultados:**

As mulheres conceberam a amamentação como um ato de impacto na saúde da criança e promotor de vínculo, todos resultados esperados. O aconselhamento foi fundamental para que superassem as dúvidas, os medos e os desafios da amamentação. Essa relação de suporte foi uma experiência que mobilizou dimensões da esperança e repercutiu na memória afetiva.

**Conclusão:**

A relação com a aconselhadora, componente da dimensão afiliativa da esperança, favoreceu e organizou o enfrentamento dos desafios da amamentação na direção do objeto esperançado.

## INTRODUÇÃO

A Organização Mundial da Saúde (OMS)^(1)^ e o Ministério da Saúde do Brasil^(2)^ indicam a oferta de leite humano (LH), de modo exclusivo, como a melhor forma de alimentar uma criança até os seus 6 meses de vida. Apesar das recomendações, as taxas de aleitamento exclusivo estão abaixo das indicativas^(3)^, com frequente uso de suplementos e fórmulas lácteas no Brasil e no mundo^(4)^. Menos da metade das crianças é amamentada exclusivamente até o sexto mês de vida, embora a OMS recomende esforços para que a taxa de aleitamento exclusivo alcance 60% até 2030^(5)^.

É premente apostas na promoção e na proteção da amamentação, sendo o aconselhamento em aleitamento materno (AAM) uma das possibilidades. O AAM fundamenta-se na consideração e no respeito à nutriz, aos seus desejos e às possibilidades em relação à amamentação^(6)^. O profissional aconselhador dá centralidade ao encontro e ao diálogo para o suporte à amamentação, o que requer habilidades relacionais e comunicacionais^(6)^.

Um estudo de revisão sistemática com metanálise revelou que o AAM é eficaz para o aumento da duração da amamentação exclusiva^(7)^. Assinala-se que o AAM amplia as chances de amamentação exclusiva ao longo dos seis meses de vida da criança^(8)^ e influencia a decisão e a manutenção do aleitamento no primeiro ano de vida^(9)^.

São determinantes para a decisão e a manutenção da amamentação a crença de que o LH é benéfico para a criança^(9,10)^ e a rede de apoio social, sobretudo no suporte emocional e prático à amamentação^(11)^. Informações, crenças culturais e valores pessoais e familiares atuam sobre as decisões em amamentação^(9,10)^.

No entanto, apesar de políticas públicas e discursos institucionais abordarem a amamentação, inseguranças, dificuldades, dores e solidão fazem parte da experiência das nutrizes^(12)^, e pressões sociais e culturais buscam determinar a alimentação da criança, interferindo no projeto de amamentação. Nesse contexto, a esperança emerge como um recurso que favorece o enfrentamento e a recuperação^(13)^, com indicativos de que pode ser promovida.

A esperança tem sido cada vez mais considerada nos cuidados com a saúde e a enfermagem. Tomá-la em conta na amamentação é inovador e tem o potencial de gerar discussões, sobretudo por abarcar especificidades, desejos e subjetividades^(14)^. Ademais, pode gerar indicativas ao cuidado em saúde e enfermagem, inclusive no contexto do AAM. Observa-se que a literatura sobre amamentação ainda não abordou a esperança como tema central. Não foram localizados estudos qualitativos, no contexto do AAM, que a tenham analisado. Assim, indagou-se: como mulheres que receberam AAM no alojamento conjunto (AC) experienciam a esperança? O objetivo foi de compreender a esperança na amamentação na perspectiva de mulheres que receberam AAM.

## MÉTODO

### Desenho do Estudo

A ferramenta *COnsolidated criteria for REporting Qualitative research* ^(15)^ orientou o relatório do estudo e a escrita deste artigo.

Trata-se de um estudo qualitativo com adoção do Modelo Multidimensional da Esperança^(16)^, que teoriza a esperança a partir de duas esferas (generalizada e particularizada) com as mesmas seis dimensões (afetiva, cognitiva, afiliativa, comportamental, contextual e temporal)^(16)^. A esfera particularizada difere da generalizada pela factibilidade do esperançado, atuando no *coping* construtivo^(16)^.

De acordo com o modelo adotado neste estudo, a dimensão contextual considera e assinala a influência das circunstâncias de vida sobre a esperança. Ela está relacionada à dimensão temporal, que reconhece a influência da historicidade da pessoa e a interconexão entre passado, presente e futuro. Essas dimensões se articulam com a dimensão cognitiva, relativa a desejos, intenções, conhecimentos, apreciação de intervenientes e determinantes da vida. Sensações, emoções e sentimentos integram a dimensão afetiva, e ações e comportamentos direcionados ao manejo e à consecução do esperançado constam na dimensão comportamental. As interações sociais (consigo e com outros) e seus desdobramentos à vivência fazem parte da dimensão afiliativa^(16)^.

### Local, População e Critérios de Seleção

Este estudo foi aninhado ao estudo piloto de um ensaio clínico randomizado (ECR), realizado de fevereiro a junho de 2023 em três centros de AC de maternidades de hospitais universitários localizados em Minas Gerais, Rio de Janeiro e Bahia.

As participantes deste estudo integraram o grupo de intervenção de estudo piloto de ECR, cujo objetivo foi investigar a eficácia do AAM durante a internação da díade no AC^(17)^. O grupo de intervenção recebeu de duas a quatro sessões de AAM durante a internação no AC, desenvolvidas por uma aconselhadora enfermeira capacitada em AAM (com formação teórico- prática de 76 horas). Destaca-se que pesquisadoras deste estudo não participaram da intervenção e da coleta de dados do estudo piloto de ECR.

Incluíram-se primíparas que intencionavam amamentar e que haviam integrado o grupo de intervenção do estudo piloto do ECR mencionado acima e que possuíam condições (pessoais e de estrutura técnica, destaque à internet) para se envolver em entrevista narrativa *online*.

Todas as 29 mulheres que participaram do estudo piloto do grupo intervenção foram convidadas a participar. O convite à pesquisa ocorreu no contexto das ligações de seguimento do estudo piloto, com reforço via mensagem escrita via *WhatsApp*®. Ao manifestarem interesse em participar, as convidadas receberam um termo de consentimento *online* e agendaram a entrevista para um dia e horário de sua preferência. Das 29 convidadas, seis recusaram participar (três por não possuírem condições e recursos para receber ligações por videochamada e três por não disporem de tempo para a entrevista), e 12 não responderam após três tentativas de contato das pesquisadoras. Participaram do presente estudo 11 mulheres.

### Coleta de Dados

A coleta de dados ocorreu entre maio e setembro de 2024, por meio de entrevista única, individual, semiestruturada e *online*, a partir de videochamadas na plataforma *WhatsApp*®, com audiogravação mediada por um aparelho gravador. No início da videochamada, foi solicitada à participante autorização para gravação, sendo explicada de forma clara a sua finalidade. As entrevistadoras, primeira e última autoras deste manuscrito, mantiveram suas câmeras ligadas para favorecer a interação com a participante, que teve a opção de usar ou não a câmera, escolhendo o que lhe trouxesse mais conforto.

As entrevistadoras receberam formação para entrevistas qualitativas (20 horas), ministrada pela segunda autora, pesquisadora com *expertise* em estudos qualitativos. A duração das entrevistas variou de 26 a 52 minutos, com média de 30 minutos, sendo disparada pela colocação “Conte-me como vem alimentando a sua criança desde o nascimento até os dias atuais”, seguida das proposições “Relembre os contatos com a aconselhadora em aleitamento materno. O que foi significante pra você?” e “Como esses contatos influenciaram a sua esperança e a tomada de decisão em relação à amamentação?”. Outras perguntas foram apresentadas para ampliar a compreensão do narrado.

Na nona entrevista, identificou-se que o conjunto de dados permitia a compreensão da experiência de esperança, existindo a repetição de elementos estruturantes do fenômeno, sugerindo uma boa densidade de dados^(18)^. Como faltavam apenas duas mulheres entre aquelas que demonstraram interesse em contribuir com o estudo, elas foram entrevistadas, contabilizando 11 participantes.

### Análise e Tratamento dos Dados

O processo analítico teve início com a elaboração de notas de pesquisa pela primeira e última autoras deste manuscrito após cada entrevista. Esse material subsidiou as interpretações e inferências realizadas durante a análise e discussão dos dados empíricos. Para mitigar a influência de vieses individuais e assegurar a fidedignidade da análise, adotou-se um processo de triangulação entre os autores. A primeira e a segunda autora conduziram as fases da análise de forma independente, com o suporte de pesquisadoras com pertença ao tema e ao modelo de esperança adotado (quarta, quinta e sexta autoras do manuscrito).

As audiogravações foram armazenadas em pasta no *Google Drive* da primeira e última autoras até que fossem transcritas na íntegra. As entrevistas foram transcritas com o auxílio do aplicativo *Transkriptor*® e transferidas para documento do *Microsoft Word*® pela primeira autora, sendo conferidas posteriormente na íntegra pela segunda autora. As transcrições, após conferidas pelas pesquisadoras, foram enviadas pelas participantes por meio do *WhatsApp*®, com a solicitação de ajustes, se necessário, o que não ocorreu, sendo, assim, consideradas validadas. Após a validação, as audiogravações foram excluídas da nuvem.

A análise temática^(19)^ foi o referencial metodológico adotado, conduzida pela primeira autora, sob o suporte da última autora. Inicialmente, foram feitas leituras reiteradas da transcrição das entrevistas e das anotações da pesquisa para identificar significados e padrões associados à amamentação, à esperança e à AAM. Trechos representativos (seleção de elementos chaves) foram extraídos e processados no software Atlas.ti® para agrupamento temático. Essa organização sofreu novas análises dirigidas pelo Modelo Multidimensional da Esperança^(16)^. Dessa forma, buscou-se compreender o objeto da esperança, suas manifestações, sentimentos, relações e a articulação desses elementos no processo de esperança na amamentação e no AAM. Também se buscou compreender como os aspectos da temporalidade e da historicidade, envolvendo passado, presente e futuro, se manifestavam e afetavam a esperança na amamentação. Códigos foram identificados e, posteriormente, agrupados em temas (etapas da seleção dos códigos, e do estabelecimento de temas). Os padrões e relações entre eles (etapa da conceituação) foram apreciados e conduziram ao desenvolvimento do modelo conceitual, etapa final do método^(19)^. Portanto, a análise teórica dirigida esteve adotada.

Em termos de reflexividade/posicionalidade, as pesquisadoras que conduziram a coleta e a análise de dados tinham em comum serem enfermeiras da área materno-infantil, acreditarem nos benefícios do LH e defenderem a amamentação. Em relação às diferenças, destacou-se serem ou não mães. A posicionalidade foi discutida no âmbito da formação para entrevistas qualitativas mencionada acima, aspecto que garantiu esforços para mitigar os efeitos da posicionalidade durante a coleta e análise de dados.

No processo investigativo, a reflexividade e a posicionalidade das pesquisadoras foram problematizadas por meio de discussões analíticas entre elas, registro de notas reflexivas durante a coleta e a análise de dados, e revisão crítica das categorias emergentes. Buscou-se, assim, evitar que pressupostos favoráveis à amamentação orientassem de forma acrítica a interpretação das narrativas, garantindo uma abertura analítica para a expressão de ambivalências, dificuldades ou experiências negativas relatadas pelas participantes. Esse processo permitiu que os significados atribuídos pelas mulheres à experiência investigada fossem compreendidos em sua complexidade, preservando a centralidade das vozes das participantes e reduzindo o risco de reforço de narrativas normativas pró-amamentação.

### Aspectos Éticos

Trata-se de um estudo com aprovação ética, sob Parecer n° 6.274.311 de 31 de agosto de 2023 (Certificado de Apresentação para Apreciação Ética n^o^ 4 61321122.3.1001.8667), e alinhado às Diretrizes e Normas Regulamentadoras de Pesquisas envolvendo seres humanos, contidas na Resolução Nacional n^o^ 510/2012/CNS/MS. Todas as participantes assinaram o Termo de Consentimento Livre e Esclarecido. No uso de excertos, a identificação adota a letra “M”, alusiva à mulher, seguida do número ordinal que traduz sua entrada no estudo.

## RESULTADOS

Participaram 11 mulheres, sendo quatro de Minas Gerais, quatro do Rio de Janeiro e três da Bahia. Todas as crianças filhas receberam aleitamento em algum momento da vida, sendo que a maioria (n = 7) permaneceu em amamentação exclusiva por seis meses; dessas, seis ainda mantinham a amamentação na data da entrevista. Oito participantes autodeclararam-se pardas; dez tinham mais de nove anos de estudos; e seis foram submetidas à cesárea. A idade das crianças no período da coleta de dados variou de 14 a 18 meses, com média de 15 meses.

A experiência da esperança na amamentação está apresentada a partir de dois temas: “Saúde e desenvolvimento da criança: desejo projetado no amamentar”; e “Manejo prático da amamentação e manutenção da esperança”. O Quadro 1 traz, além dos temas, os subtemas identificados.

**Quadro 1:** Codificação dos temas e subtemas derivados da análise dos dados obtidos no estudo – São Carlos, SP, Brasil, 2025.

Temas	Subtemas
Saúde e desenvolvimento da criança: desejo projetado no amamentar	Reconhecimento dos benefícios do leite humano
	Apropriar-se da amamentação
Manejo prático da amamentação e manutenção da esperança	Empatia e julgamento de pessoas da rede social
	Enfrentar as dificuldades sob altruísmo
	Singularidade da relação com a criança na amamentação
	Mutualidade na relação com a aconselhadora

Identificou-se o desejo das mulheres de amamentar seus filhos, na esperança de lhes proporcionar o melhor alimento, criar laços afetivos fortes e favorecer sua saúde e desenvolvimento. O altruísmo moral mostrou-se central para que elas se mantivessem esperançosas com a amamentação e para que fizessem renúncias e suportassem o “custo emocional” de suas decisões sobre a amamentação. Foram constantemente desafiadas a remodelar a esperança.

### Tema: Saúde e Desenvolvimento da Criança: Desejo Projetado no Amamentar

Todas as participantes esperançam amamentar em função do “reconhecimento dos benefícios do LH” para a criança, o que conduz ao “apropriar-se da amamentação”, comportamento impulsionado e mantido pelo compromisso moral com a criança e pelo seu papel social de nutriz. Ressaltaram a contribuição de outras nutrizes no manejo de sofrimentos e dificuldades. O compartilhamento de saberes e experiencias delas atuou nos diálogos internalizados para ampliarem a autonomia e terem *insights* na busca pelo sucesso prático na amamentação.

### Subtema: Reconhecimento dos Benefícios do Leite Humano

O LH foi reconhecido como o melhor alimento para a saúde de crianças, conduzindo a esforços para garantir sua oferta à criança: *Eu falei! Eu não quero dar fórmula porque sei o quanto a amamentação é essencial para todo desenvolvimento* (M7 – desmame ao sexto mês); [...] *o leite materno não tem outro igual, a criança cresce saudável* (M3 – em aleitamento (14 meses), AME até o sexto mês); [...] *o que me fez não desistir da amamentação* [...] *foi a importância do leite, do bem que ele faz para a saúde, previne doenças* (M4 – em aleitamento (14 meses), AME até o sexto mês).

Interseccionalidades endossaram a busca: [...] *o leite que vem de graça. O gasto é mínimo* [...] *eu sou mãe solo, então fiz a opção pela amamentação exclusiva até por uma questão econômica também, além dos benefícios que ela traz para a criança* (M5 – em aleitamento (16 meses), AME até o sexto mês).

Esse subtema assinalou as dimensões cognitiva e comportamental da esperança, estruturando a busca e a persistência no alcance da amamentação. Aspectos da socialização ampliada remetem às dimensões contextual, temporal e afiliativa da esperança, dinamizadas com o objetivo de mantê-la durante a amamentação.

### Subtema: Apropriar-se da Amamentação

A necessidade de apropriarem-se da amamentação e ampliar conhecimentos (teóricos e práticos) se intensificou diante do nascimento da criança: *Eu queria aprender mais, apesar do tanto que eu já tinha visto antes na gravidez. Depois que a menina nasceu, eu queria aprender mais ainda para ficar mais preparada* (M3 – em aleitamento (14 meses), AME até o sexto mês). A concretude da criança e as interações com ela reforçam a intenção de ofertar LH via amamentação sob um evidente altruísmo moral.

A complexidade e a multifatorialidade da amamentação são ponderadas e relativizadas, assim como a esperança: [...] *porque cada mãe fala um relato da amamentação.* [...] *outras tiveram uma amamentação bem tranquila, outras tiveram um período curto,* [...] *tem criança que acaba não pegando o peito*, [...] *e eu ficava pensando se seria assim comigo também* (M8 – desmame aos dois meses).

Enfrentaram as adversidades com o apoio de profissionais e de pessoas próximas, sobretudo da família. Essas experiências relacionais são resgatadas nas conversas internalizadas, promovendo força interior e esperança: *Foi muito importante eu tentar. Porque, no meio desse desafio de ser mãe de primeira viagem, do puerpério,* [...] *eu fiz compromisso esse comigo. Eu não vou ir para fórmula.* [...] *por ter tido esse acompanhamento das enfermeiras e tal, eu falei para mim: “A gente vai passar”. Foram 15 dias muito difíceis. Meu peito feriu, mas eu falei, vamos continuar* (M7 – desmame ao sexto mês).

A amamentação, um projeto voltado para o futuro, é construído no presente a partir da remodelação de sua esperança, envolvendo as dimensões cognitiva, contextual, temporal, afiliativa e afetiva da esperança.

### Tema: Manejo Prático da Amamentação e Manutenção da Esperança

Esse tema abrange os desdobramentos da “empatia e do julgamento de pessoas da rede social” para decisões sobre amamentação, perpassa abdicações e impõe o “enfrentamento das dificuldades sob altruísmo” no manejo da amamentação, vivenciado e sustentado na “singularidade da relação com a criança durante a amamentação” e na “mutualidade na relação com a aconselhadora”, ambos relevantes para o enfrentamento e a manutenção da esperança, considerando as particularidades de seu contexto e experiência.

### Subtema: Empatia e Julgamento de Pessoas da Rede Social

As relações sociais e consigo mesmas são determinantes da esperança. Elas trouxeram, simultaneamente, experiências de empatia e orgulho, julgamentos, sofrimentos e abnegação. São experiências que envolvem as dimensões contextual e afiliativa e que contribuem para a conformação da esperança particularizada, em contraponto à esperança generalizada.

Encontrar relações promotoras e sustentadoras de sua persistência na amamentação contribuiu para o enfrentamento dos intervenientes, sobretudo nos tempos iniciais. Destacaram-se as manifestações explícitas e implícitas do companheiro/pai da criança, reconhecendo seus esforços e apostas na amamentação, assim como as de pessoas significativas: *Ele* (referindo-se ao companheiro) *via meu sofrimento com a amamentação, ficou do meu lado.* [...] *falava que ia passar e ali eu me agarrava* (M3 – em aleitamento (14 meses), AME até o sexto mês); *Mãe, eu acho que eu não dou conta, não.* [...] *eu vou lá no pediatra, vou pedir para ele passar um leite* [...] *aí ela falou: “Você vai dar leite industrializado se você tem leite suficiente? Aguenta a dorzinha, pensa que é por amor a ela que você vai passar por isso” (*M4 – em aleitamento (14 meses), AME até o sexto mês); [...] *ela* (mãe da participante) *falou que sentiu a minha dor quando assou meu mamilo* (M9 – em aleitamento (17 meses), AME até ao sexo mês). A mãe da nutriz esteve destacada pelas falas de motivação e pelas manifestações de sororidade.

De modo concomitante, manejaram interações que desestimulavam seu caminhar na direção da amamentação: *Alguns amigos falam assim: “Você está se sacrificando, dá a fórmula, toda criança toma fórmula”* (M7 – desmame ao sexto mês); *Ah, essa menina está com fome* [...] *compra logo um leite.* [...] *comecei a ficar nervosa, acabei não indo no pediatra e comecei a complementar com a fórmula por conta* [...] *coloquei uma mamadeira e a menina começou a largar meu peito* (M11 – desmame aos dois meses).

### Subtema: Enfrentar as Dificuldades Sob Altruísmo

Dificuldades no manejo prático da amamentação levaram as participantes a relativizar a concepção de que a amamentação é um processo intrínseco e natural, sem entraves: *Na minha cabeça, tipo assim, amamentar vai ser aquela coisa linda. Na minha cabeça, seria assim. Quando não foi, eu fiquei bem mal, porque eu queria muito* […] (M2 – desmame no primeiro mês).

A insuficiente apresentação ou pouca tematização das dificuldades, problemas e desafios relativos à amamentação foi denunciada, e a intervenção se mostrou uma esperança e um enfrentamento: *Eu vejo gente romantizando a amamentação como se ela fosse uma coisa assim, um mel, e não é! Não é!* (ênfase). *Tem lá os seus desafios, tem o seu momento difícil* [...] *você não vê a mulher com olheira, não vê a mulher acordada de madrugada* [...] *o vídeo que mostram, o neném está sempre dormindo, a casa está sempre organizada, o menino nunca chora,* [...] *então, isso é complicado, porque a gente vê muita coisa e, na realidade, não é bem assim* (M4 – em aleitamento (14 meses), AME até o sexto mês).

No início da amamentação, as experiências dolorosas diante das lesões no complexo aréolo-mamilar trouxeram sofrimento e o desejo de desistir, impondo a si sofrimentos: *Os 15 primeiros dias foram bem complicados. Quando dava a hora dela mamar, assim, eu já sentia* [...] *porque o peito doía* [...] *eu não vou mentir, deu vontade de desistir* (M3 – em aleitamento (14 meses), AME até o sexto mês); *Quando a* (nome da criança) *estava com uns 40 dias, o meu peito rachou, todos os dois.* [...] *quando racha, dói, sangra* [...] *dá aquele desespero* [...] *sofri um mês* [...] *mas eu não desisti, minha filha mamou com sangue, com dor, eu chorando, mordendo toalha* (M4 – em aleitamento (14 meses), AME até o sexto mês). No hiato do desistir e persistir, esteve o altruísmo moral voltado à oferta do melhor alimento à criança.

As experiências álgica e de não dormir foram amplamente mencionadas, como experiências ruins justificadas para si como atos em prol da criança, entendimento que as levou a se esforçarem para seguir em frente: *Meu peito ficou ferido, mas, para mim, não me importava. Às vezes, eu nem sentia a dor* [...] *o que eu sabia é que ali era bom para minha filha. A importância do leite materno na vida de minha filha e o que poderia fazer lá na frente, quando ela teve maior, por ela ter sido amamentada* (M11 – desmame aos dois meses); *Até hoje, eu não consegui dormir uma noite de sono* [...] (M7 – desmame ao sexto mês).

O cuidado de si, atividades de lazer e laborais ficaram em um patamar secundário: *Fiquei mais descuidada comigo, porque não tenho tempo para nada* [...] (pausou estudos e passeios) *por não querer deixá-lo sozinho, fico com medo dele passar fome sem o meu peito* (M1 – em aleitamento (14 meses), misto desde o quarto mês); [...] *eu pedi até para sair do CLT, durante a gravidez, para poder ficar o mais próximo possível dele, para não ele acabar saindo do peito logo* (M8 – desmame aos dois meses).

O retorno ao trabalho, intercorrências puerperais e a hospitalização da criança impuseram limites à amamentação e induziram o replanejamento ou o abandono da prática: *Fiquei muito brava* (introdução de mamadeira e chupeta pela avó), *mas enfim, eu não falei nada, porque precisava de ajuda para trabalhar* (M1 – em aleitamento (14 meses), misto desde o quarto mês); *O* (nome da criança) *teve uma bronquiolite, e precisou ficar internado*. [...] *ficamos 18 dias internados em isolamento. Ele desaprendeu a mamar, ficou na sonda.* [...] *eu que fiquei insistindo, eu não queria desistir* [...] (M8 – desmame aos dois meses).

Todas as participantes, por tempos distintos, buscaram resistir a intervenientes promotores do desmame: *O que eu queria mesmo era estar amamentando até agora* (um ano e cinco meses), *infelizmente aconteceu isso dele acabar desmamando, mas eu aproveitei bastante, não me arrependo até hoje, não, de tudo que enfrentei* (M8 – desmame aos dois meses); *Mesmo que o aleitamento foi misto, foi muita, nossa luta.* [...] *não foi por falta de esforço.* [...] *eu entendi ser um esforço válido* (M11 – desmame aos dois meses).

A projeção de uma amamentação sem dificuldades, frequentemente romantizada em vídeos, contrapôs-se ao vivido. Ancoradas sobretudo nas dimensões cognitiva (o conhecimento dos benefícios) e afetiva (o vínculo afetivo), foram movidas a sustentar a sua esperança pela amamentação.

### Subtema: Singularidade da Relação com A Criança na Amamentação

A mulher teceu e fortaleceu a sintonia e o elo com a sua criança, aspecto de sentido único, motivador do amamentar e da remodelação da esperança*: É um momento único só nosso* (dela e da criança) [...] *ver o olhinho, o jeito de felicidade que ele olha para mim quando dou de “mamá”, o jeito que ele se acalma* (M1 – em aleitamento (14 meses), misto desde o quarto mês); *É uma coisa assim, inexplicável* [...] *ele me olhando, colocava a mãozinha, me alisava. Então, eu amava* (M9 – em aleitamento (17 meses), AME até ao sexto mês).

As dimensões afetiva e afiliativa da esperança estiveram retratadas como amor, prazer, vínculo, interação e conexão com a criança, ampliando o sentido da amamentação para além dos benefícios do LH.

### Subtema: Mutualidade na Relação com A Aconselhadora

O AAM foi percebido como um suporte, sobretudo nos momentos iniciais. A atitude da aconselhadora enfrentou o medo, o sofrimento e as dúvidas, trazendo tranquilidade: *Ela* (aconselhadora) *foi muito essencial para minha jornada de amamentação. Se não fosse ela, talvez eu não conseguisse* (M1 – em aleitamento (14 meses), misto desde o quarto mês); *Só de saber que você não está sozinha ali, que tem uma pessoa para tentar te ajudar* (M2 – desmame no primeiro mês); *Eu falei assim para* (nome da aconselhadora) [...] *eu não tenho leite. E ela: “Não, fica tranquila, você tem, sim* (leite)*”, e isto acalma* (M7 – desmame ao sexto mês).

A aconselhadora mediou os entendimentos e ofertou intervenções práticas com especificidade para cada nutriz: [...] *aquela primeira instrução fez toda a diferença.* [...] *porque, na prática mesmo, se você não tiver uma pessoa ali para te aconselhar, pra te auxiliar, não vai, não* (M4 – em aleitamento (14 meses), AME até o sexto mês); *Foi a minha primeira experiência como mãe. Estava naquele momento apreensivo, me ajudou bastante a tirar minhas* dúvidas (M11 – desmame aos dois meses); *Eu tenho um mamilo invertido, então eu estava tendo um pouco de dificuldade também na pega da criança. E com a orientação dela, eu consegui fazer com que minha filha mamasse* (M11 – desmame aos dois meses).

A presença e a comunicação da aconselhadora foram diferenciais e ficam na memória: *Ela vai ficar na minha lembrança para sempre assim. Acho que no dia em que eu tiver outro filho, vou logo lembrar dela, a questão da amamentação* (M3 – em aleitamento (14 meses), AME até o sexto mês); *A minha decisão de ter ido à frente com a amamentação partiu por um diálogo, algo que me marcou muito. Então, a maneira de acolher* [...] *ela mudou toda a minha dinâmica com a minha filha* (M7 – desmame ao sexto mês).

A Figura 1 apresenta o modelo conceitual, os temas e os subtemas revelados neste estudo. O desejo de amamentar a criança se articula com o prover saúde à criança. Assim, ocorre o “enfrentar as dificuldades sob altruísmo”, envolvendo a “empatia e julgamento de pessoas da rede social”. O processo vai revelando a “singularidade da relação com a criança na amamentação” e esteve sustentado pela “mutualidade na relação com a aconselhadora”. O modelo conceitual amplia a compressão e permite a aplicação prática do Modelo Multidimensional de Esperança na experiencia específica de amamentação.

**Figura 1:** Modelo conceitual da esperança na amamentação: vivência de mulheres que receberam aconselhamento em aleitamento materno.

## DISCUSSÃO

A amamentação foi concebida como algo impactante para a saúde da criança e promotora do vínculo com ela, aspectos desejados e esperados. Assim, os envolvidos se empenharam na amamentação, na esperança de alcançá-la, enfrentando os desafios. A esperança favoreceu transformações de sentidos e percursos ao longo da busca pela manutenção da amamentação. Especificamente a aconselhadora foi promotora da esperança particularizada, intervindo, sobretudo, nas dimensões cognitiva e afetiva da esperança, consolidando-se como um elemento da dimensão afiliativa.

A literatura endossa a relação da amamentação com a saúde da criança e o vínculo^(20)^, assim como assinala a urgência do suporte informacional, seja ele individual, coletivo ou ampliado, como nas campanhas de amamentação. Este estudo revelou ter o suporte informacional efeitos na esperança na amamentação, promovendo motivação e engajamento, atuando no *agency thinking* ^(21)^. O AAM aferiu um suporte personalíssimo e foi essencial para a esperança na amamentação.

Contextos de incertezas, com ameaças ao controle, como é a amamentação, requerem esperança para enfrentamentos alinhados aos intervenientes particulares. Apropriar-se da amamentação, comportamento almejado pelas participantes, denotou a busca por controle e a intenção de autoeficácia. O ato de apropriar envolve as dimensões cognitiva, afiliativa, comportamental e contextual da esperança^(16)^.

Uma forma do profissional favorecer apropriação é estimular a partilha das vivências na amamentação entre gestantes e nutrizes, relativizando as possibilidades e os contextos. O escutar as experiências promove o contato com os participantes da amamentação e possibilita o manejo deles^(22)^, considerando a singularidade de cada vivência de amamentação^(23)^. A mediação profissional deve abordar os envolvidos na amamentação, dando concretude às suas preocupações, dúvidas e incertezas, considerando o particular de cada situação. Particularizar é essencial para um enfrentamento construtivo, pois permite uma ponderação crítica e contextualizada^(16)^.

Ademais, apostar na curadoria e indicação de sites, vídeos e outras mídias digitais sobre amamentação é promissor, pois oportuniza a apropriação do tema e combate a disseminação de construções sociais romantizadas, que foram identificadas como obstáculos para a amamentação.

O manejo prático da amamentação tem, na empatia das pessoas da rede social, um recurso. O cuidado centrado na família destaca a urgência dos profissionais em considerarem a família como demandante de cuidados, assim como a influência da dinâmica familiar sobre os enfrentamentos individuais e vice-versa^(24)^, aspecto reforçado neste estudo. Reconhecer e considerar a família, nas pessoas significativas para cada um que amamenta, é uma intervenção que promove a esperança na amamentação. Relações intrafamiliares fortalecem a esperança quando estão sustentadas pela confiança, pelo esforço de ajuda e pela escuta atenta^(24)^.

A influência da crença no leite fraco e o questionamento sobre a valia dos esforços para amamentar foram identificados e endossam outros estudos^(25)^, revelando as permanências de construções sociais e a dificuldade em contrapô-las. As crenças pertencem às dimensões cognitiva e contextual, impactando muito as dimensões afetiva e cognitiva da esperança^(16)^. Elas estão fortemente relacionadas ao desmame^(26)^.

Dificuldades e sofrimentos existiram, e o enfrentamento ocorreu sob fragilidades emocionais, inseguranças e dores físicas (sobretudo as decorrentes de lesões no complexo aréolo- mamilar), questões pouco ou nada abordadas no processo de amamentação oferecido por profissionais, conforme denunciado pelas participantes. É essencial avançar na abordagem voltada à promoção e à proteção da amamentação^(14)^. Nesse sentido, utilizar o Modelo Multidimensional da Esperança nas intervenções de suporte em amamentação favorece situar e intervir sobre os intervenientes, lidar com sentidos e remodelar comportamentos.

No hiato empatia-altruísmo, estão as concepções motivacionais de altruísmo e egoísmo, com destaque para o egoísmo exclusivo e os desdobramentos da preocupação empática na produção da motivação altruísta^(27)^. A preocupação empática refere-se à emoção voltada ao outro, desencadeada e congruente com o bem-estar percebido. O altruísmo, no hiato egoísmo-altruísmo, refere-se a um estado motivacional com o objetivo final de aumentar o bem-estar do outro. Nesse contexto, a motivação altruísta, induzida pela empatia, faz parte da parentalidade humano. O altruísmo induzido pela empatia não é inerentemente positivo ou negativo; ele simplesmente existe^(27)^ e esteve presente neste estudo.

O trabalho, as intercorrências puerperais e a hospitalização da criança afetaram a amamentação e lhe conferiram particularidades. No entanto, todas essas situações não se constituem justificativas para o desmame, apesar de comumente levá-lo a ocorrer^(28)^. Para todas elas, planos terapêuticos que envolvam o Banco de Leite Humano são essenciais^(29)^, apesar de não terem surgido nos resultados desta pesquisa. Indicam-se estudos futuros sobre a relação do equipamento com a esperança na amamentação.

As contribuições da amamentação para o vínculo e o apego estão amplamente descritas^(20)^, foram percebidas pelas participantes e sustentaram sua esperança na amamentação. A partir dessa constatação, dialogar sobre a relação com a criança durante a amamentação pode evidenciar e validar nuances dessa relação, destacando olhares e toques, ambos reforçados em nossos resultados como promotores da esperança. Ademais, o modelo conceitual do presente estudo guarda aproximações com a Teoria Interativa de Amamentação^(23)^, com destaques para o objetivo de alcançar a amamentação e a centralidade da interação para a busca da amamentação. Em outras palavras, a mulher interage com a criança e com os outros sistemas interpessoais e sociais na direção da amamentação. Sentidos, crenças e memórias vinculados à sua inserção social e à historicidade são dinamizados. Este estudo acrescenta ao modelo mencionado^(23)^ a evidência da potência do construto esperança e de suas dimensões na compreensão dos participantes e em seu enfrentamento.

As participantes ressaltaram que contaram com o AAM e assinalaram suas contribuições para as diversas dimensões da esperança, sobretudo a afiliativa, a cognitiva, a comportamental e a afetiva. No entanto, percebe-se que houve desdobramentos para as dimensões contextual e temporal. Os preceitos do AAM, que apostam no diálogo e na centralidade da pessoa, alinham-se aos estruturantes do uso intencional da esperança nas intervenções em saúde. A revelação de acolhimento e gratidão como desfechos da relação são evidências disto e estão alinhadas a um cuidado integral, humano, equânime e promotor de esperança.

As limitações deste estudo estão relacionadas ao número reduzido de participantes, todas primíparas, em três centros hospitalares, o que não permite a generalização e a transferibilidade dos resultados. Porém, estudos qualitativos não têm essa intenção. Além disso, como as participantes aceitaram integrar o estudo piloto de ECR em AAM, pode-se inferir que elas já tinham inclinação para a amamentação. Outra ressalva refere- se ao fato de serem mulheres do grupo de intervenção, ou seja, que receberam AAM, o que pode gerar vieses de “gratidão” em relação à aconselhadora, bem como de desejabilidade social em relação à amamentação. Esse conjunto representa uma limitação operacional e analítica. Por outro lado, ressalta-se que o objetivo foi compreender a esperança na amamentação, recorte que posicionou o foco dos pesquisadores na esperança. De todo modo, indicam-se estudos com quem interrompeu a amamentação precocemente, não recebeu AAM ou não tinha projeto ou inclinação para o aleitamento, a fim de avançar as evidências.

As entrevistas realizadas remotamente podem ser consideradas fragilidades por reduzirem pistas não verbais. Reforça-se a importância de desenvolver novos estudos com entrevistas presenciais. Contudo, enfatiza-se que o fato de serem remotas facilitou a abordagem das participantes.

O estudo também possui como limitação o possível viés recordatório, uma vez que as entrevistas ocorreram em média 15 meses após as mulheres receberem o AAM. O tempo decorrido entre a intervenção e a entrevista pode comprometer a riqueza interpretativa relacionada à memória. No entanto, acredita-se que isso foi minimizado devido à profundidade e à densidade dos relatos.

Como contribuição do estudo, observou-se, através das narrativas, que a esperança ajuda na amamentação e o aconselhamento sustenta a esperança. A esperança organiza o enfrentamento da amamentação; o objeto esperançado é projetado na criança, e não na mulher; o sofrimento é legitimado moralmente pelo altruísmo; a relação com a aconselhadora, componente da dimensão afiliativa da esperança, favoreceu e organizou o enfrentamento dos desafios da amamentação na direção do objeto esperançado.

## CONCLUSÃO

A amamentação está relacionada ao desejo de proporcionar à criança uma vida saudável e de criar laços e conexões únicas e duradouras com ela, o que motiva a busca e a esperança na amamentação, o enfrentamento dos desafios e a persistência no processo.

Para obter maior êxito prático na amamentação, as participantes se envolveram com a apropriação dela, mas denunciaram o discurso romantizado, que reverbera negativamente, sobretudo na dimensão afetiva da esperança. Ademais, na dimensão afiliativa, elas destacaram a importância de integrar os julgamentos da rede social e a empatia. Entre outros aspectos, manejaram crenças, experiências dolorosas e abdicações para amamentar. Nas relações mais proximais, sobretudo com a parceria/pai da criança e com a mãe, elas encontraram acolhimento, assim como com a aconselhadora. Essas relações se compuseram com a conexão singular provida pela relação com a criança, que tem um papel diferenciado na esperança de amamentar e é central para a mobilização no enfrentamento das inúmeras situações ao longo da trajetória da amamentação.

A partir dos resultados, destacam-se as seguintes recomendações para a promoção da esperança na amamentação: explorar a relação com a criança e com a rede familiar; realizar a curadoria de tecnologias educacionais e de cuidados em amamentação, evitando a romantização do processo; instaurar diálogos sobre o trabalho e a amamentação; investir em estratégias educacionais e de cuidados coletivas; e estimular a participação de parceiros significativos da mulher no pré-natal. Porém, sobretudo, é preciso investir na relação personalíssima com a nutriz e sua família, com base no modelo conceitual da esperança na amamentação alcançado neste estudo.

## Data Availability

Todo o conjunto de dados que dá suporte aos resultados deste estudo está disponível mediante solicitação ao autor correspondente.

# Translation: Esperanza en la lactancia materna frente al consejo sobre lactancia: un análisis cualitativo a la luz del Modelo Multidimensional de la Esperanza

**DOI:** 10.1590/1980-220X-REEUSP-2025-0394es

**Authors:** Allison Scholler de Castro Villas Boas, Monika Wernet, Mariana Torreglosa Ruiz, Aline Oliveira Silveira, Zaida Charepe, Ana Maria Linares, Jéssica Aparecida da Silva

**Affiliations:** 1 Universidade Federal de São Carlos, Programa de Pós-Graduação em Enfermagem, São Carlos, SP, Brasil.; 2 Universidade Federal do Triângulo Mineiro, Programa de Pós-Graduação em Atenção à Saúde, Uberaba, MG, Brasil.; 3 Universidade de Brasília, Brasília, DF, Brasil.; 4 Universidade Católica Portuguesa, Centro de Investigação Interdisciplinar em Saúde, Lisboa, Portugal.; 5 University of Kentucky, College of Nursing, Lexington, Estados Unidos.

**Keywords:** Lactancia Materna, Esperanza, Consejo, Destete, Investigación Cualitativa

## Abstract

**Objetivo:**

Comprender la esperanza en la lactancia materna desde la perspectiva de las mujeres que recibieron consejo sobre lactancia.

**Método:**

Investigación cualitativa integrada en un ensayo clínico, basada en un análisis temático guiado por el Modelo Multidimensional de la Esperanza. Once mujeres, de entre 14 y 18 meses después del parto, todas participantes en un ensayo clínico piloto, recibieron consejo sobre lactancia materna en la unidad de alojamiento conjunto de hospitales de maternidad ubicados en Minas Gerais, Rio de Janeiro y Bahía, Brasil. Se realizó una única entrevista semiestructurada grabada en audio entre mayo y septiembre de 2024.

**Resultados:**

Las mujeres percibieron la lactancia materna como un acto de gran impacto en la salud del niño y un promotor del vínculo afectivo, resultados que coincidían con las expectativas. El consejo fue fundamental para ayudarlas a superar dudas, miedos y dificultades relacionadas con la lactancia materna. Esta relación de apoyo movilizó dimensiones de esperanza y resonó en su memoria emocional.

**Conclusión:**

la relación con el consejero, un componente de la dimensión afiliativa de la esperanza, favoreció y organizó el afrontamiento de los desafíos de la lactancia materna hacia el objetivo deseado.

## INTRODUCCIÓN

La Organización Mundial de la Salud (OMS)^(1)^ y el Ministerio de Salud de Brasil^(2)^ recomiendan la alimentación exclusiva con leche materna como la mejor forma de alimentar a un bebé hasta los 6 meses de vida. A pesar de las recomendaciones, las tasas de lactancia materna exclusiva están por debajo de las indicadas^(3)^, con un uso frecuente de suplementos y fórmulas lácteas en Brasil y en el mundo^(4)^. Menos de la mitad de los niños son amamantados exclusivamente hasta el sexto mes de vida, aunque la OMS recomienda esfuerzos para que la tasa de lactancia materna exclusiva alcance el 60% para el 2030^(5)^.

Es urgente apostar por la promoción y la protección de la lactancia materna, siendo el asesoramiento sobre lactancia materna (ALM) una de las opciones. El ALM se basa en la consideración y el respeto hacia la madre lactante, sus deseos y sus posibilidades en relación con la lactancia materna^(6)^. El profesional que brinda consejo le da prioridad al encuentro y al diálogo para apoyar la lactancia materna, lo que requiere habilidades relacionales y comunicativas^(6)^.

Un estudio de revisión sistemática con metanálisis reveló que el ALM es eficaz para aumentar la duración de la lactancia materna exclusiva^(7)^. Cabe destacar que el ALM aumenta las posibilidades de lactancia materna exclusiva a lo largo de los primeros seis meses de vida del niño^(8)^ e influye en la decisión y el mantenimiento de la lactancia durante el primer año de vida^(9)^.

Son factores determinantes para la decisión y el mantenimiento de la lactancia materna la creencia de que la leche materna es beneficiosa para el niño^(9,10)^ y la red de apoyo social, sobre todo en cuanto al apoyo emocional y práctico a la lactancia^(11)^. La información, las creencias culturales y los valores personales y familiares influyen en las decisiones sobre la lactancia^(9,10)^.

Sin embargo, a pesar de que las políticas públicas y los discursos institucionales abordan la lactancia materna, las inseguridades, las dificultades, el dolor y la soledad forman parte de la experiencia de las madres lactantes^(12)^, y las presiones sociales y culturales buscan determinar la alimentación del niño, interfiriendo en el proyecto de lactancia. En este contexto, la esperanza surge como un recurso que favorece la superación y la recuperación^(13)^, con indicios de que puede promoverse.

La esperanza se ha tomado cada vez más en cuenta en la atención de la salud y la enfermería. Considerarla en el contexto de la lactancia materna es innovador y tiene el potencial de generar debates, sobre todo porque abarca particularidades, deseos y subjetividades^(14)^. Además, puede aportar indicaciones para la atención en salud y la enfermería, incluso en el contexto de la ALM. Se observa que la literatura sobre lactancia materna aún no ha abordado la esperanza como tema central. No se encontraron estudios cualitativos, en el contexto de la ALM, que la hayan analizado. Por lo tanto, se planteó la siguiente pregunta: ¿cómo experimentan la esperanza las mujeres que recibieron ALM en el alojamiento conjunto (AC)? El objetivo fue comprender la esperanza en la lactancia materna desde la perspectiva de las mujeres que recibieron ALM.

## MÉTODO

### Diseño del Estudio

La herramienta *COnsolidated criteria for REporting Qualitative research* ^(15)^ sirvió de guía para el informe del estudio y la redacción de este artículo.

Se trata de un estudio cualitativo que adopta el Modelo Multidimensional de la Esperanza^(16)^, el cual teoriza sobre la esperanza a partir de dos esferas (generalizada y particularizada) con las mismas seis dimensiones (afectiva, cognitiva, afiliativa, conductual, contextual y temporal)^(16)^. La esfera particularizada se diferencia de la generalizada por la viabilidad de lo esperado, lo cual influye en el *coping (enfrentamiento)* constructivo^(16)^.

De acuerdo con el modelo adoptado en este estudio, la dimensión contextual considera y destaca la influencia de las circunstancias de la vida sobre la esperanza. Está relacionada con la dimensión temporal, que reconoce la influencia de la historicidad de la persona y la interconexión entre el pasado, el presente y el futuro. Estas dimensiones se articulan con la dimensión cognitiva, relacionada con los deseos, las intenciones, los conocimientos y la valoración de los agentes y los factores determinantes de la vida. Las sensaciones, emociones y sentimientos forman parte de la dimensión afectiva, mientras que las acciones y comportamientos orientados al manejo y la consecución de lo esperado se incluyen en la dimensión conductual. Las interacciones sociales (con uno mismo y con los demás) y sus repercusiones en la experiencia forman parte de la dimensión afiliativa^(16)^.

### Lugar, Población y Criterios de Selección

Este estudio se realizó como parte de un estudio piloto de un ensayo clínico randomizado (ECR), llevado a cabo de febrero a junio de 2023 en tres centros de alojamiento conjunto de maternidades de hospitales universitarios ubicados en Minas Gerais, Río de Janeiro y Bahía.

Las participantes de este estudio formaron parte del grupo de intervención del estudio piloto del ECR, cuyo objetivo fue investigar la eficacia del ALM durante la hospitalización de la díada en el AC^(17)^. El grupo de intervención recibió de dos a cuatro sesiones de ALM durante la hospitalización en la UC, impartidas por una enfermera consejera capacitada en ALM (con formación teórico-práctica de 76 horas). Cabe destacar que las investigadoras de este estudio no participaron en la intervención ni en la recolección de datos del estudio piloto del ECR.

Se incluyeron primíparas que tenían la intención de amamantar y que habían formado parte del grupo de intervención del estudio piloto del ECR mencionado anteriormente, y que contaban con las condiciones (personales y de infraestructura técnica, en especial acceso a Internet) para participar en una entrevista narrativa en línea.

Se invitó a participar a las 29 mujeres que habían formado parte del grupo de intervención en el estudio piloto. La invitación a participar en la investigación se realizó en el marco de las llamadas de seguimiento del estudio piloto, con un recordatorio mediante mensaje escrito a través de *WhatsApp*®. Al manifestar su interés en participar, las invitadas recibieron un formulario de consentimiento en línea y programaron la entrevista para el día y la hora de su preferencia. De las 29 invitadas, seis se negaron a participar (tres porque no contaban con las condiciones ni los recursos para recibir videollamadas y tres porque no disponían de tiempo para la entrevista), y 12 no respondieron tras tres intentos de contacto por parte de las investigadoras. Participaron en el presente estudio 11 mujeres.

### Recolección de Datos

La recolección de datos se llevó a cabo entre mayo y septiembre de 2024, mediante una entrevista única, individual, semiestructurada y en línea, a través de videollamadas en la plataforma *WhatsApp*®, con grabación de audio realizada mediante un dispositivo de grabación. Al inicio de la videollamada, se solicitó a la participante autorización para la grabación, explicándole claramente su propósito.

Las entrevistadoras, la primera y la última autoras de este manuscrito, mantuvieron sus cámaras encendidas para facilitar la interacción con la participante, quien tuvo la opción de usar o no su cámara, eligiendo lo que le resultara más cómodo.

Las entrevistadoras recibieron capacitación para entrevistas cualitativas (20 horas), impartida por la segunda autora, investigadora con experiencia en estudios cualitativos. La duración de las entrevistas varió entre 26 y 52 minutos, con un promedio de 30 minutos, y se inició con la pregunta «Cuénteme cómo ha estado alimentando a su bebé desde el nacimiento hasta la actualidad», seguida de las preguntas «¿Qué recuerda de sus contactos con la consejeraen lactancia materna? ¿Qué fue significativo para usted?» y «¿Cómo influyeron esos contactos en su esperanza y en la toma de decisiones con respecto a la lactancia materna?». Se plantearon otras preguntas para ampliar la comprensión de lo narrado.

En la novena entrevista, se identificó que el conjunto de datos permitía comprender la experiencia de la esperanza, ya que se observaba la repetición de elementos estructurantes del fenómeno, lo que sugería una buena densidad de datos^(18)^. Como solo faltaban dos mujeres entre aquellas que habían mostrado interés en contribuir al estudio, se las entrevistó, con lo que el total de participantes ascendió a 11.

### Análisis y Tratamiento de los Datos

El proceso analítico comenzó con la elaboración de notas de investigación por parte de la primera y la última autoras de este manuscrito después de cada entrevista. Este material sirvió de base para las interpretaciones e inferencias realizadas durante el análisis y la discusión de los datos empíricos. Para mitigar la influencia de sesgos individuales y garantizar la fiabilidad del análisis, se adoptó un proceso de triangulación entre las autoras. La primera y la segunda autora llevaron a cabo las fases del análisis de manera independiente, con el apoyo de investigadoras especializadas en el tema y en el modelo de esperanza adoptado (cuarta, quinta y sexta autoras del manuscrito).

Las grabaciones de audio se almacenaron en una carpeta en *Google Drive* de la primera y la última autora hasta que se transcribieron en su totalidad. Las entrevistas fueron transcritas con la ayuda de la aplicación *Transkriptor*® y transferidas a un documento de *Microsoft Word*® por la primera autora, siendo posteriormente revisadas en su totalidad por la segunda autora. Las transcripciones, tras ser revisadas por las investigadoras, fueron enviadas a las participantes a través de *WhatsApp*®, con la solicitud de que realizaran ajustes, si fuera necesario, lo cual no ocurrió, por lo que se consideraron validadas. Tras la validación, las grabaciones de audio se eliminaron de la nube.

Se adoptó como marco metodológico el análisis temático^(19)^, llevado a cabo por la primera autora con el apoyo de la última autora. Inicialmente, se realizaron lecturas repetidas de la transcripción de las entrevistas y de las notas de la investigación para identificar significados y patrones asociados a la lactancia materna, la esperanza y la ALM. Se extrajeron fragmentos representativos (selección de elementos clave) y se procesaron en el software Atlas.ti® para su agrupación temática. Esta organización fue sometida a nuevos análisis guiados por el Modelo Multidimensional de la Esperanza^(16)^. De esta manera, se buscó comprender el objeto de la esperanza, sus manifestaciones, sentimientos, relaciones y la articulación de estos elementos en el proceso de esperanza en la lactancia materna y en la ALM. También se buscó comprender cómo los aspectos de la temporalidad y la historicidad, que abarcan el pasado, el presente y el futuro, se manifestaban y afectaban la esperanza en la lactancia materna. Se identificaron códigos y, posteriormente, se agruparon en temas (etapas de selección de códigos y de establecimiento de temas). Se evaluaron los patrones y las relaciones entre ellos (etapa de conceptualización), lo que condujo al desarrollo del modelo conceptual, etapa final del método^(19)^. Por lo tanto, se adoptó el análisis teórico dirigido.

En términos de reflexividad y posicionalidad, las investigadoras que llevaron a cabo la recolección y el análisis de datos tenían en común ser enfermeras del área materno-infantil, creer en los beneficios de la leche materna y defender la lactancia materna. En cuanto a las diferencias, se destacó el hecho de ser o no madres. La posicionalidad se discutió en el marco de la capacitación para entrevistas cualitativas mencionada anteriormente, aspecto que garantizó esfuerzos para mitigar los efectos de la posicionalidad durante la recolección y el análisis de datos.

En el proceso de investigación, la reflexividad y la posicionalidad de las investigadoras se cuestionaron mediante discusiones analíticas entre ellas, el registro de notas reflexivas durante la Recolección y el análisis de datos, y la revisión crítica de las categorías emergentes. De esta manera, se buscó evitar que los supuestos a favor de la lactancia materna orientaran de manera acrítica la interpretación de las narrativas, garantizando una apertura analítica para la expresión de ambivalencias, dificultades o experiencias negativas relatadas por las participantes. Este proceso permitió comprender en toda su complejidad los significados que las mujeres atribuían a la experiencia investigada, preservando la centralidad de las voces de las participantes y reduciendo el riesgo de reforzar narrativas normativas a favor de la lactancia materna.

### Aspectos Éticos

Se trata de un estudio con aprobación ética, conforme al Dictamen n.º 6.274.311 del 31 de agosto de 2023 (Certificado de Presentación para Evaluación Ética n.º 4 61321122.3.1001.8667), y se ajusta a las Directrices y Normas Reguladoras de las Investigaciones con seres humanos, contenidas en la Resolución Nacional n.º 510/2012/CNS/MS. Todas las participantes firmaron el Formulario de Consentimiento Libre e Informado. En los extractos, la identificación utiliza la letra «M», que alude a la mujer, seguida del número ordinal que indica su participación en el estudio.

## RESULTADOS

Participaron 11 mujeres, cuatro de Minas Gerais, cuatro de Río de Janeiro y tres de Bahía. Todas las hijas recibieron lactancia materna en algún momento de su vida, y la mayoría (n = 7) permaneció en lactancia materna exclusiva durante seis meses; de ellas, seis aún seguían amamantando al momento de la entrevista. Ocho participantes se autodefinieron como de raza mestiza; diez tenían más de nueve años de estudios; y seis se sometieron a cesárea. La edad de los niños en el momento de la recolección de datos osciló entre los 14 y los 18 meses, con un promedio de 15 meses.

La experiencia de la esperanza en la lactancia materna se presenta a partir de dos temas: «Salud y desarrollo del niño: deseo proyectado en la lactancia materna»; y «Manejo práctico de la lactancia materna y mantenimiento de la esperanza».

La Tabla 1 presenta, además de los temas, los subtemas identificados.

**Tabla 1:** Codificación de los temas y subtemas derivados del análisis de los datos obtenidos en el estudio – São Carlos, SP, Brasil, 2025.

Temas	Subtemas
Salud y desarrollo infantil: deseo proyectado en la lactancia materna	Reconocimiento de los beneficios de la leche materna
	Apropiarse de la lactancia materna
Manejo práctico de la lactancia materna y mantenimiento de la esperanza	Empatía y juicios de las personas de la red social
	Enfrentar las dificultades con altruismo
	Singularidad de la relación con el niño durante la lactancia
	Reciprocidad en la relación con la consejera

Se identificó el deseo de las mujeres de amamantar a sus hijos, con la esperanza de brindarles la mejor alimentación, crear fuertes lazos afectivos y favorecer su salud y desarrollo. El altruismo moral resultó ser fundamental para que mantuvieran la esperanza en la lactancia materna y para que hicieran sacrificios y soportaran el «costo emocional» de sus decisiones al respecto. Se vieron constantemente desafiadas a renovar su esperanza.

### Tema: Salud y Desarrollo Infantil: Deseo Proyectado en la Lactancia Materna

Todas las participantes esperan amamantar debido al «reconocimiento de los beneficios de la leche materna» para el niño, lo que las lleva a «apropiarse de la lactancia materna», un comportamiento impulsado y sostenido por el compromiso moral con el niño y por su rol social como madres lactantes. Destacaron la contribución de otras madres lactantes en el manejo de los sufrimientos y las dificultades. El intercambio de conocimientos y experiencias de estas madres influyó en los diálogos internalizados para ampliar su autonomía y obtener *perspectivas* en la búsqueda del éxito práctico en la lactancia materna.

### Subtema: Reconocimiento de los Beneficios de la Leche Materna

La leche materna fue reconocida como el mejor alimento para la salud de los niños, lo que dio lugar a esfuerzos para garantizar su suministro al niño: *¡Ya lo dije! No quiero darle fórmula porque sé lo esencial que es la lactancia materna para todo el desarrollo* (M7 – destetada al sexto mes); [...] *la leche materna no tiene igual, el niño crece sano* (M3 – en lactancia (14 meses), amamantada hasta el sexto mes); [...] *lo que me hizo no dejar de amamantar* [...] *fue la importancia de la leche, lo bien que le hace a la salud, previene enfermedades* (M4 – en lactancia (14 meses), lactancia materna exclusiva hasta los seis meses).

Las interseccionalidades respaldaron la búsqueda: [...] *la leche que viene gratis. El gasto es mínimo* [...] *soy madre soltera, así que opté por la lactancia materna exclusiva también por una cuestión económica, además de los beneficios que le brinda al niño* (M5 – en lactancia (16 meses), lactancia materna exclusiva hasta el sexto mes).

Este subtema destacó las dimensiones cognitiva y conductual de la esperanza, estructurando la búsqueda y la persistencia para lograr la lactancia materna. Los aspectos de la socialización ampliada remiten a las dimensiones contextual, temporal y de afiliación de la esperanza, dinamizadas con el objetivo de mantenerla durante la lactancia.

### Subtema: Apropiarse de la Lactancia Materna

La necesidad de apropiarse de la lactancia materna y ampliar los conocimientos (teóricos y prácticos) se intensificó ante el nacimiento de la niña: *Quería aprender más, a pesar de todo lo que ya había visto antes durante el embarazo. Después de que naciera la niña, quería aprender aún más para estar mejor preparada* (M3 – en lactancia materna (14 meses), AME hasta el sexto mes). La presencia real del bebé y las interacciones con él refuerzan la intención de ofrecer leche materna a través de la lactancia, impulsada por un evidente altruismo moral.

La complejidad y la multifactorialidad que influyen en la lactancia se sopesan y relativizan, al igual que la esperanza: [...] *porque cada mamá cuenta su propia historia de lactancia.* [...] *otras tuvieron una lactancia muy tranquila, otras tuvieron un período corto,* [...] *hay niños que al final no se agarran al pecho*, [...] *y yo me quedaba pensando si a mí también me pasaría lo mismo* (M8 – destete a los dos meses).

Enfrentaron las adversidades con el apoyo de profesionales y de personas cercanas, sobre todo de la familia. Estas experiencias relacionales se reflejan en las conversaciones interiorizadas, lo que fomenta la fortaleza interior y la esperanza: *Fue muy importante que lo intentara. Porque, en medio de ese desafío de ser madre primeriza, del puerperio,* [...] *me comprometí conmigo misma. No voy a recurrir a la leche de fórmula.* [...] *gracias al apoyo de las enfermeras y demás, me dije a mí misma: “Vamos a superar esto”. Fueron 15 días muy difíciles. Me dolían los senos, pero me dije: “Vamos a seguir”* (M7 – destete al sexto mes).

La lactancia materna, un proyecto orientado hacia el futuro, se construye en el presente a partir de la remodelación de su esperanza, involucrando las dimensiones cognitiva, contextual, temporal, afiliativa y afectiva de la esperanza.

### Tema: Manejo Práctico de la Lactancia Materna y Mantenimiento de la Esperanza

Este tema abarca las repercusiones de la «empatía y el juicio de las personas del entorno social» en las decisiones sobre la lactancia materna, atraviesa las renuncias e impone el «enfrentamiento de las dificultades con altruismo» en el manejo de la lactancia materna, vivido y sostenido en la «singularidad de la relación con el niño durante la lactancia» y en la «reciprocidad en la relación con la asesora», ambos aspectos relevantes para enfrentar y mantener la esperanza, considerando las particularidades de su contexto y experiencia.

### Subtema: Empatía y Juicios de las Personas de la Red Social

Las relaciones sociales y con ellas mismas son determinantes de la esperanza. Estas trajeron, simultáneamente, experiencias de empatía y orgullo, juicios, sufrimientos y abnegación. Son experiencias que involucran las dimensiones contextual y afiliativa y que contribuyen a la conformación de la esperanza particularizada, en contrapunto a la esperanza generalizada.

Encontrar relaciones que promovieran y sostuvieran su persistencia en la lactancia materna contribuyó a que las participantes pudieran afrontar el reto, sobre todo en los primeros tiempos. Se destacaron las manifestaciones explícitas e implícitas de la pareja o el papá del niño, reconociendo sus esfuerzos y su compromiso con la lactancia materna, así como las de personas significativas: *Él* (refiriéndose a la pareja) *veía mi sufrimiento con la lactancia materna, se mantuvo a mi lado.* [...] *me decía que ya pasaría y ahí me aferraba* (M3 – en lactancia (14 meses), lactancia materna exclusiva hasta el sexto mes); *Mamá, creo que no puedo con esto, no.* [...] *voy al pediatra, le voy a pedir que me recete leche* [...] *entonces ella dijo: «¿Vas a darle leche de fórmula si tienes suficiente leche? Aguanta el dolorcito, piensa que es por amor a ella que vas a pasar por esto» (*M4 – en lactancia (14 meses), lactancia materna exclusiva hasta el sexto mes); [...]*ella* (la mamá de la participante) *dijo que sintió mi dolor cuando se me enrojeció el pezón* (M9 – en lactancia (17 meses), lactancia materna exclusiva hasta el sexto mes). La madre de la lactante se destacó por sus comentarios motivadores y sus muestras de solidaridad entre mujeres.

Al mismo tiempo, tuvieron que lidiar con interacciones que desalentaban su camino hacia la lactancia materna: *Algunos amigos dicen así: “Te estás sacrificando, dale fórmula, todos los niños toman fórmula”* (M7 – destetada al sexto mes); *Ay, esta niña tiene hambre* [...] *compra ya una leche.* [...]*empecé a ponerme nerviosa, al final no fui al pediatra y comencé a complementar con fórmula por mi cuenta*[...]*le di un biberón y la niña empezó a soltar mi pecho* (M11 – destete a los dos meses).

### Subtema: Enfrentar las Dificultades con Altruismo

Las dificultades en el manejo práctico de la lactancia llevaron a las participantes a relativizar la idea de que la lactancia es un proceso intrínseco y natural, sin obstáculos: *En mi cabeza, más o menos, amamantar iba a ser algo hermoso. En mi cabeza, sería así. Cuando no fue así, me sentí muy mal, porque lo deseaba mucho* […] (M2 – desmame al primer mes).

Se denunció la insuficiente presentación o la escasa discusión de las dificultades, los problemas y los desafíos relacionados con la lactancia materna, y la intervención se reveló como una esperanza y una forma de enfrentarlos: *Veo a gente idealizando la lactancia materna como si fuera algo así, una delicia, ¡y no lo es! ¡No lo es!* (énfasis). *Tiene sus desafíos, tiene sus momentos difíciles* [...] *no ves a la mujer con ojeras, no ves a la mujer despierta en la madrugada* [...] *en el video que muestran, el bebé siempre está durmiendo, la casa siempre está ordenada, el niño nunca llora,* [...]*entonces, eso es complicado, porque vemos muchas cosas y, en realidad, no es así* (M4 – en lactancia (14 meses), lactancia materna exclusiva hasta el sexto mes).

Al inicio de la lactancia, las experiencias dolorosas debido a las lesiones en el complejo areola-pezón le causaron sufrimiento y el deseo de rendirse, imponiéndose a sí misma ese sufrimiento: *Los primeros 15 días fueron muy complicados. Cuando llegaba la hora de que ella tomara, ya sentía* [...] *porque me dolía el pecho* [...] *no voy a mentir, tuve ganas de rendirme* (M3 – en lactancia (14 meses), lactancia materna exclusiva hasta el sexto mes); *Cuando la* (nombre del niño) *tenía unos 40 días, se me agrietaron ambos pechos.* [...] *cuando se agrietan, duelen, sangran* [...] *te entra esa desesperación* [...] *sufrí un mes* [...] *pero no me rendí, mi hija mamó con sangre, con dolor, yo llorando, mordiéndome una toalla* (M4 – en lactancia (14 meses), lactancia materna exclusiva hasta el sexto mes). Entre la disyuntiva de rendirse o persistir, se encontraba el altruismo moral orientado a ofrecerle al niño el mejor alimento.

Las experiencias de dolor y de falta de sueño se mencionaron ampliamente, como experiencias negativas justificadas para ellas mismas como actos en beneficio de la niña, una comprensión que las llevó a esforzarse por seguir adelante: *Me dolían los pechos, pero a mí no me importaba. A veces, ni siquiera sentía el dolor* [...] *lo que sabía es que eso era bueno para mi hija. La importancia de la leche materna en la vida de mi hija y lo que podría significar en el futuro, cuando ella creciera, por haber sido amamantada* (M11 – destete a los dos meses); *Hasta el día de hoy, no he podido dormir una noche completa* [...] (M7 – desteteal sexto mes).

El cuidado personal, las actividades de ocio y laborales quedaron en un segundo plano: *Me descuidé más a mí misma, porque no tengo tiempo para nada* [...] (dejó de lado los estudios y las salidas) *porque no quería dejarlo solo, me da miedo que pase hambre sin mi pecho* (M1 – en lactancia (14 meses), mixta desde el cuarto mes); [...] *incluso pedí dejar mi empleo de tiempo completo durante el embarazo para poder estar lo más cerca posible de él, para que no terminara dejando el pecho tan pronto* (M8 – destete a los dos meses).

El regreso al trabajo, las complicaciones posparto y la hospitalización del niño impusieron límites a la lactancia materna e indujeron a replantear o abandonar la práctica: *Me enojé mucho* (introducción del biberón y el chupón por parte de la abuela), *pero bueno, no dije nada, porque necesitaba ayuda para trabajar* (M1 – en lactancia materna (14 meses), mixta desde el cuarto mes); *El* (nombre del niño) *tuvo una bronquiolitis y tuvo que ser hospitalizado*. [...] *estuvimos 18 días hospitalizados en aislamiento. Desaprendió a mamar, lo alimentaron por sonda.* [...] *fui yo quien insistí, no quería rendirme* [...] (M8 – destete a los dos meses).

Todas las participantes, durante distintos períodos, intentaron resistirse a quienes promovían el destete: *Lo que realmente quería era seguir amamantando hasta ahora* (un año y cinco meses), *lamentablemente pasó que él terminó de destetarse, pero lo disfruté mucho, no me arrepiento hasta el día de hoy, no, de todo lo que enfrenté* (M8 – destete a los dos meses); *Aunque la lactancia fue mixta, fue una gran lucha para nosotras.* [...]*no fue por falta de esfuerzo.*[...]*entendí que fue un esfuerzo válido* (M11 – destete a los dos meses).

La idea de una lactancia sin dificultades, a menudo idealizada en los videos, contrastaba con lo vivido. Basándose sobre todo en las dimensiones cognitiva (el conocimiento de los beneficios) y afectiva (el vínculo afectivo), se sintieron motivadas a mantener su esperanza en la lactancia.

### Subtema: Singularidad de la Relación con el Niño Durante la Lactancia

La mujer tejió y fortaleció la sintonía y el vínculo con su hijo, un aspecto único y significativo, que motivó la lactancia y la renovación de la esperanza*: Es un momento único, solo nuestro* (de ella y del niño) [...] *ver sus ojitos, la forma tan feliz en que me mira cuando le doy el “pecho”, la forma en que se calma* (M1 – en lactancia (14 meses), mixta desde el cuarto mes); *Es algo así, inexplicable* [...] *él mirándome, ponía su manita, me acariciaba. Entonces, yo lo amaba* (M9 – en lactancia (17 meses), lactancia materna exclusiva hasta el sexto mes).

Las dimensiones afectivas y de afiliación de la esperanza se describieron como amor, placer, vínculo, interacción y conexión con el niño, ampliando el significado de la lactancia materna más allá de los beneficios de la leche materna.

### Subtema: Reciprocidad en la Relación con la Consejera

La ALM fue percibida como un apoyo, sobre todo en los momentos iniciales. La actitud de la consejera hizo frente al miedo, al sufrimiento y a las dudas, aportando tranquilidad: *Ella* (la asesora) *fue fundamental en mi proceso de lactancia. Si no fuera por ella, tal vez no hubiera podido* (M1 – en lactancia materna (14 meses), mixta desde el cuarto mes); *Solo saber que no estás sola ahí, que hay una persona que intenta ayudarte* (M2 – destete en el primer mes); *Le dije esto a* (nombre de la asesora) [...] *no tengo leche. Y ella: “No, quédate tranquila, sí que tienes* (leche)*”, y eso te tranquiliza* (M7 – destetada al sexto mes).

La consejera facilitó el entendimiento y ofreció intervenciones prácticas específicas para cada madre lactante: [...] *esa primera indicación marcó toda la diferencia.* [...] *porque, en la práctica, si no tienes a alguien ahí para aconsejarte, para ayudarte, no va a funcionar* (M4 – en lactancia (14 meses), LME hasta el sexto mes); *Fue mi primera experiencia como mamá. En ese momento estaba nerviosa, y me ayudó mucho a aclarar mis* dudas (M11 – destete a los dos meses); *Tengo un pezón invertido, así que también tenía un poco de dificultad para que la niña se prendiera. Y con su orientación, logré que mi hija tomara* (M11 – destete a los dos meses).

La presencia y la comunicación de la consejera marcaron la diferencia y se me quedaron grabadas: *La recordaré así para siempre. Creo que el día que tenga otro hijo, enseguida me acordaré de ella, del tema de la lactancia* (M3 – en lactancia (14 meses), LME hasta el sexto mes); *Mi decisión de seguir adelante con la lactancia surgió de una conversación, algo que me marcó mucho. Entonces, la forma en que me acogió* [...] *cambió por completo mi dinámica con mi hija* (M7 – destetada al sexto mes).

La Figura 1 presenta el modelo conceptual, los temas y los subtemas revelados en este estudio. El deseo de amamantar al niño se articula con el cuidado de su salud. Así, se da el «enfrentamiento de las dificultades desde el altruismo», lo cual implica la «empatía y el juicio de las personas de la red social». El proceso va revelando la «singularidad de la relación con el niño durante la lactancia» y se vio respaldado por la «reciprocidad en la relación con la asesora». El modelo conceptual amplía la comprensión y permite la aplicación práctica del Modelo Multidimensional de la Esperanza en la experiencia específica de la lactancia materna.

**Figura 1:** Modelo conceptual de la esperanza en la lactancia materna: experiencias de mujeres que recibieron asesoramiento sobre lactancia materna.

## DISCUSIÓN

La lactancia materna se concibió como algo que tiene un impacto significativo en la salud del niño y que fomenta el vínculo con él, aspectos deseados y esperados. Por lo tanto, las personas involucradas se esforzaron por lograr la lactancia materna, con la esperanza de alcanzarla, enfrentando los desafíos. La esperanza favoreció transformaciones de significado y trayectorias a lo largo de la búsqueda por mantener la lactancia materna. Específicamente, la asesora fue promotora de una esperanza personalizada, interviniendo, sobre todo, en las dimensiones cognitiva y afectiva de la esperanza, consolidándose como un elemento de la dimensión afiliativa.

La literatura respalda la relación entre la lactancia materna y la salud del niño y el vínculo^(20)^, así como señala la urgencia del apoyo informativo, ya sea individual, colectivo o ampliado, como en las campañas de lactancia materna. Este estudio reveló que el apoyo informativo tiene efectos en la esperanza en la lactancia materna, promoviendo la motivación y el compromiso, y actuando en el *agency thinking* ^(21)^. El ALM evaluó un apoyo muy personalizado y fue esencial para la esperanza en la lactancia materna.

Los contextos de incertidumbre, con amenazas al control, como es el caso de la lactancia materna, requieren esperanza para enfrentar situaciones de manera alineada con los actores particulares. La apropiación de la lactancia materna, comportamiento anhelado por las participantes, denotó la búsqueda de control y la intención de autoeficacia. El acto de apropiarse involucra las dimensiones cognitiva, afiliativa, conductual y contextual de la esperanza^(16)^.

Una forma en que el profesional puede favorecer la apropiación es estimular el intercambio de experiencias sobre la lactancia entre mujeres embarazadas y madres lactantes, relativizando las posibilidades y los contextos. Escuchar las experiencias promueve el contacto con las personas involucradas en la lactancia materna y permite abordarlas^(22)^, tomando en cuenta la singularidad de cada experiencia de lactancia^(23)^. La mediación profesional debe dirigirse a quienes participan en la lactancia materna, dando concreción a sus preocupaciones, dudas e incertidumbres, considerando las particularidades de cada situación. La personalización es esencial para un enfoque constructivo, ya que permite una reflexión crítica y contextualizada^(16)^.

Además, apostar por la selección y recomendación de sitios web, videos y otros medios digitales sobre la lactancia materna es prometedor, ya que brinda la oportunidad de apropiarse del tema y combate la difusión de constructos sociales idealizados, que se han identificado como obstáculos para la lactancia materna.

El manejo práctico de la lactancia materna cuenta con un recurso: la empatía de las personas de la red social. El cuidado centrado en la familia resalta la urgencia de que los profesionales consideren a la familia como receptora de cuidados, así como la influencia de la dinámica familiar sobre los desafíos individuales y viceversa^(24)^, un aspecto reforzado en este estudio. Reconocer y tomar en cuenta a la familia, en las personas significativas para cada madre que amamanta, es una intervención que promueve la esperanza en la lactancia materna. Las relaciones intrafamiliares fortalecen la esperanza cuando se sustentan en la confianza, el esfuerzo por ayudar y la escucha atenta^(24)^.

Se identificó la influencia de la creencia en la “leche débil” y el cuestionamiento sobre el valor de los esfuerzos por amamantar, lo cual concuerda con otros estudios^(25)^, revelando la persistencia de construcciones sociales y la dificultad para contrarrestarlas. Las creencias pertenecen a las dimensiones cognitiva y contextual, y tienen un gran impacto en las dimensiones afectiva y cognitiva de la esperanza^(16)^. Están estrechamente relacionadas con el destete^(26)^.

Hubo dificultades y sufrimiento, y la experiencia se vivió en medio de fragilidad emocional, inseguridades y dolores físicos (sobre todo los derivados de lesiones en el complejo areola-pezón), cuestiones que apenas o en absoluto se abordaron en el proceso de lactancia ofrecido por los profesionales, según denunciaron las participantes. Es esencial avanzar en un enfoque orientado a la promoción y protección de la lactancia materna^(14)^. En este sentido, utilizar el Modelo Multidimensional de la Esperanza en las intervenciones de apoyo a la lactancia materna permite contextualizar e intervenir con los participantes, abordar los significados y remodelar los comportamientos.

En la brecha entre la empatía y el altruismo se encuentran las concepciones motivacionales del altruismo y el egoísmo, con especial énfasis en el egoísmo exclusivo y las ramificaciones de la preocupación empática en la generación de la motivación altruista^(27)^. La preocupación empática se refiere a la emoción dirigida hacia el otro, desencadenada y congruente con el bienestar percibido. El altruismo, en la brecha entre egoísmo y altruismo, se refiere a un estado motivacional cuyo objetivo final es aumentar el bienestar del otro. En este contexto, la motivación altruista, inducida por la empatía, forma parte de la parentalidad en los seres humanos. El altruismo inducido por la empatía no es inherentemente positivo ni negativo; simplemente existe^(27)^ y estuvo presente en este estudio.

El trabajo de parto, las complicaciones posparto y la hospitalización del niño afectaron la lactancia materna y le dieron características particulares. Sin embargo, ninguna de estas situaciones constituye una justificación para el destete, aunque comúnmente conduzcan a que este ocurra^(28)^. Para todas ellas, son esenciales los planes terapéuticos que involucren al Banco de Leche Humana^(29)^, aunque no hayan surgido en los resultados de esta investigación. Se recomiendan estudios futuros sobre la relación entre el equipo y la esperanza en la lactancia materna.

Las contribuciones de la lactancia materna al vínculo y al apego están ampliamente descritas^(20)^, fueron percibidas por las participantes y sustentaron su esperanza en la lactancia materna. A partir de esta constatación, dialogar sobre la relación con el niño durante la lactancia materna puede poner de manifiesto y validar matices de dicha relación, destacando las miradas y los toques, ambos reforzados en nuestros resultados como promotores de la esperanza. Además, el modelo conceptual del presente estudio presenta similitudes con la Teoría Interactiva de la Lactancia Materna^(23)^, con énfasis en el objetivo de lograr la lactancia materna y en la centralidad de la interacción para la búsqueda de la lactancia materna. En otras palabras, la mujer interactúa con el bebé y con otros sistemas interpersonales y sociales con el fin de lograr la lactancia materna. Se dinamizan los significados, las creencias y los recuerdos vinculados a su inserción social y a su historicidad. Este estudio aporta al modelo mencionado^(23)^ la evidencia del poder del constructo «esperanza» y de sus dimensiones en la comprensión de las participantes y en su forma de afrontar la situación.

Las participantes destacaron que contaron con el ALM y señalaron sus contribuciones a las diversas dimensiones de la esperanza, sobre todo la afiliativa, la cognitiva, la conductual y la afectiva. Sin embargo, se percibe que hubo ramificaciones hacia las dimensiones contextual y temporal. Los principios del ALM, que apuestan por el diálogo y la centralidad de la persona, se alinean con los fundamentos del uso intencional de la esperanza en las intervenciones de salud. La manifestación de acogida y gratitud como resultados de la relación son evidencia de ello y están alineadas con una atención integral, humana, equitativa y promotora de la esperanza.

Las limitaciones de este estudio están relacionadas con el número reducido de participantes, todas primerizas, en tres centros hospitalarios, lo que no permite la generalización ni la transferibilidad de los resultados. Sin embargo, los estudios cualitativos no tienen esa intención. Además, dado que las participantes aceptaron formar parte del estudio piloto de ECR sobre ALM, se puede inferir que ya tenían una inclinación hacia la lactancia materna. Otra salvedad se refiere al hecho de que se trata de mujeres del grupo de intervención, es decir, que recibieron ALM, lo que puede generar sesgos de «gratitud» hacia la asesora, así como de deseabilidad social en relación con la lactancia materna. Este conjunto representa una limitación operativa y analítica. Por otro lado, cabe destacar que el objetivo fue comprender las expectativas respecto a la lactancia materna, enfoque que centró la atención de los investigadores en las expectativas. De todos modos, se recomienda realizar estudios con mujeres que interrumpieron la lactancia materna prematuramente, que no recibieron ALM o que no tenían planes ni inclinación hacia la lactancia materna, con el fin de ampliar la evidencia.

Las entrevistas realizadas de manera remota pueden considerarse una limitación, ya que reducen las señales no verbales. Se reitera la importancia de desarrollar nuevos estudios con entrevistas presenciales. Sin embargo, se destaca que el hecho de que fueran remotas facilitó el acercamiento a las participantes.

El estudio también presenta como limitación el posible sesgo de recuerdo, ya que las entrevistas se realizaron, en promedio, 15 meses después de que las mujeres recibieran el ALM. El tiempo transcurrido entre la intervención y la entrevista puede comprometer la riqueza interpretativa relacionada con la memoria. No obstante, se cree que esto se minimizó debido a la profundidad y la densidad de los relatos.

Como contribución del estudio, se observó, a través de las narrativas, que la esperanza ayuda en la lactancia materna y que el asesoramiento sustenta la esperanza. La esperanza organiza la forma de afrontar la lactancia materna; el objeto de esperanza se proyecta en el niño, y no en la mujer; el sufrimiento se legitima moralmente mediante el altruismo; la relación con la asesora, componente de la dimensión afiliativa de la esperanza, favoreció y organizó la forma de afrontar los desafíos de la lactancia materna en dirección al objeto de esperanza.

## CONCLUSIÓN

La lactancia materna está relacionada con el deseo de brindarle al niño una vida saludable y de crear vínculos y conexiones únicas y duraderas con él, lo que motiva la búsqueda y la esperanza en la lactancia materna, la superación de los desafíos y la persistencia en el proceso.

Para lograr un mayor éxito práctico en la lactancia materna, las participantes se involucraron en su apropiación, pero denunciaron el discurso idealizado, que tiene repercusiones negativas, sobre todo en la dimensión afectiva de la esperanza. Además, en la dimensión afectiva, destacaron la importancia de integrar los juicios de la red social y la empatía. Entre otros aspectos, gestionaron creencias, experiencias dolorosas y renuncias para poder amamantar. En las relaciones más cercanas, sobre todo con la pareja o el padre del niño y con la madre, encontraron acogida, al igual que con la asesora. Estas relaciones se complementaron con la conexión única que brinda la relación con el niño, quien desempeña un papel diferenciado en la esperanza de amamantar y es fundamental para la movilización a la hora de enfrentar las innumerables situaciones a lo largo del proceso de lactancia materna.

A partir de los resultados, se destacan las siguientes recomendaciones para promover la esperanza en la lactancia materna: explorar la relación con el niño y con la red familiar; seleccionar cuidadosamente las tecnologías educativas y de cuidados relacionados con la lactancia, evitando la idealización del proceso; iniciar diálogos sobre el trabajo y la lactancia materna; invertir en estrategias educativas y de cuidados colectivas; y fomentar la participación de las parejas de las mujeres durante el período prenatal. Sin embargo, sobre todo, es necesario invertir en la relación muy personal con la madre lactante y su familia, basándose en el modelo conceptual de la esperanza en la lactancia materna obtenido en este estudio.

## Data Availability

El conjunto de datos que respaldan las conclusiones de este estudio está disponible previa solicitud al autor correspondiente.
